# A Review on Machine Learning Approaches for Network Malicious Behavior Detection in Emerging Technologies

**DOI:** 10.3390/e23050529

**Published:** 2021-04-25

**Authors:** Mahdi Rabbani, Yongli Wang, Reza Khoshkangini, Hamed Jelodar, Ruxin Zhao, Sajjad Bagheri Baba Ahmadi, Seyedvalyallah Ayobi

**Affiliations:** 1 School of Computer Science and Engineering, Nanjing University of Science and Technology, Nanjing 210094, China; phd.cruxinzhao@njust.edu.cn (R.Z.); s.bagheri@njust.edu.cn (S.B.B.A.); S.valyallahayobi@njust.edu.cn (S.A.); 2 Center for Applied Intelligent Systems Research (CAISR), Halmstad University, 30118 Halmstad, Sweden; reza.khoshkangini@hh.se; 3 Faculty of Computer Science, Dalhousie University, Halifax, NS B3H 4R2, Canada; jelodar@dal.ca

**Keywords:** machine learning, classifier systems, malicious behavior detection systems, dataset, data pre-processing

## Abstract

Network anomaly detection systems (NADSs) play a significant role in every network defense system as they detect and prevent malicious activities. Therefore, this paper offers an exhaustive overview of different aspects of anomaly-based network intrusion detection systems (NIDSs). Additionally, contemporary malicious activities in network systems and the important properties of intrusion detection systems are discussed as well. The present survey explains important phases of NADSs, such as pre-processing, feature extraction and malicious behavior detection and recognition. In addition, with regard to the detection and recognition phase, recent machine learning approaches including supervised, unsupervised, new deep and ensemble learning techniques have been comprehensively discussed; moreover, some details about currently available benchmark datasets for training and evaluating machine learning techniques are provided by the researchers. In the end, potential challenges together with some future directions for machine learning-based NADSs are specified.

## 1. Introduction

Cyberattacks and network security threats have dramatically increased in emerging technologies such as Cloud, Fog, Edge computing and Internet of Things (IoT). These attacks are able to penetrate network-related environments, Cloud-based servers, and damage economic source information [1]. Network anomaly detection systems (NADSs) play an essential role in every network defense system as they monitor network packets to prevent potential threats and users’ behavioral abnormalities.

Throughout the last decade, the number of malicious incidents have dramatically increased, and such an issue leads into significant consequences for individual users, organizations, and companies. As an example, a denial-of-service (DoS) attack disorganizes the regular traffic in the network by flooding with a massive volume of traffic. Likewise, distributed denial-of-service (DDoS) targets normal network traffic by injecting a flood of network traffic attacks [2]. Zero-day attack detection is also very challenging since the template or signature specification for these types of attacks is not available [3].

**Figure 1:**
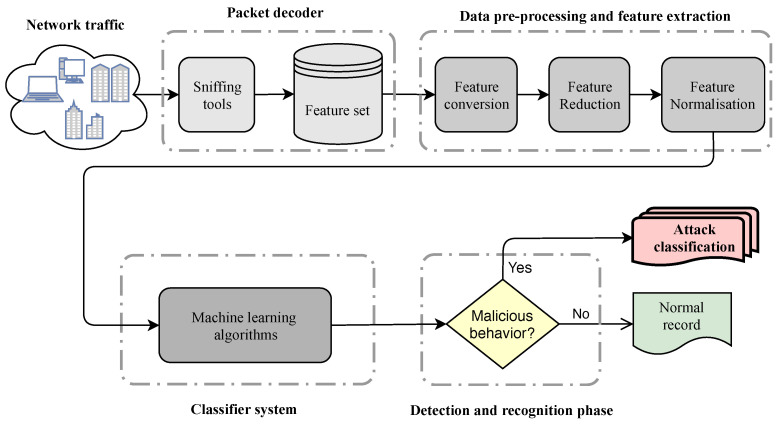
Main modules in machine learning classifier systems.

Figure 1 shows a conventional architecture of a machine learning-based network anomaly detection system, which is constructed by four main modules. The modules are itemized as follows:

- Packet decoder: this module receives raw network traffic packets and transfers suitable information to the pre-processing module.
- Pre-processing: this module receives a portion of network traffic features and prepares corresponding normalized feature vectors, which is necessary for learning-based systems in the detection module.
- Classifier system: the responsibility of this module is to build a model on top of the prepared data which discriminates malicious instances against normal ones.
- Detection and recognition: two phases are defined in this module—(i) detects the malicious instances as a binary decision problem (e.g., 0 for normal and 1 for malicious) and transmits an alert to a system administrator for making a reaction; and (ii) after any malicious behavior detection, the system can recognize various types of abnormality (attack classification).

In this study, the authors focused on the main parts and properties of network anomaly detection systems, in particular, the detection phase. Numerous machine learning methods for network malicious behavior detection have been discussed towards designing an intelligent NADS which is capable of detecting known and zero-day attacks in high-speed network traffic.

### 1.1. Related Surveys

Over the last decade, there have been various survey articles in the literature which aimed at reviewing different types of IDSs and NADSs with varied objectives. Consequently, the present article is oriented towards the main findings of some recently published surveys in the field of IDSs and particularly anomaly detection systems; in other words, it deals with the aforementioned previous findings’ merits and drawbacks. In addition, Table 1 comparatively shows the novelty and superiority of a proposed survey against recently published survey articles in the area of anomaly detection systems. Hindy et al. [4] proposed a survey about different IDS techniques and also network threats. The survey mainly focused on different types of IDSs, datasets and threats, but the authors did not cover different types of detection techniques, particularly machine learning approaches. In another study, Lu et al. [5] conducted a survey about deep learning techniques for malware intrusion detection and prediction. Although the authors provided deep learning techniques in IDSs and briefly mentioned other malware classification methods, the authors did not discuss a pre-processing step—which is an important phase in IDSs and can negatively affect the overall time complexity of a detection system. Moreover, the survey lacks a detailed classification about the variety of recently used shallow learning approaches in IDSs.

Igino et al. [6] provided a survey on adversarial attacks against IDSs on safety-critical environments. The authors provided a general taxonomy of attack tactics against IDSs and divided the detection strategy into three different phases: measurement, classification, and responses. The main challenging issue found in the detection engine in IDS techniques is implementing non-learning-based techniques to deal with the complexity of contemporary intrusions and malicious instances. Hodo et al. [7] discussed shallow and deep networks for IDSs, and the authors provided an overview of the general classification of IDSs and taxonomy with recent and past works. By comparing different learning-based techniques, they justified that the convolutional neural network (CNN) has not been exploited in the field of intrusion detection, however, they have proved that it is a good classifier. In addition, signature-based techniques are used commercially, however, the main drawback of these techniques is that they fail to detect all types of malicious instances due to not having their signature list in database. Aburomman et al. [8] conducted a survey on IDSs using ensemble and hybrid learning systems. The authors highlighted two main categories of multiple-classifier systems, homogeneous ensembles (single classification approach) and heterogeneous ensembles (two or more different classification approaches). They proved that heterogeneous approaches based on weighted majority voting are rarely implemented for IDSs and meta-heuristic optimization techniques deserve more attention based on extracted patterns in NSL-KDD dataset. Ahmed et al. [9] provided a survey of anomaly detection techniques in the financial domain, the survey mainly focused on clustering as an unsupervised learning technique to detect fraud and anomalous data against normal ones. Different types of financial fraudulent activities such as break-in fraud, billing fraud, illegal redistribution fraud, failed logins, and the issue of the scarcity of real data have been discussed throughout their article. Buczak et al. [10] proposed a survey of data mining and machine learning techniques for cybersecurity IDSs. Both misuse and anomaly detection techniques have been discussed based on important criteria such as accuracy, complexity, time for classifying an unknown instance with a trained model, and the understandability of the final solution. The biggest gap that the authors observed was the availability of labeled data, which is a very important issue when the anomaly detection and recognition phase is based on supervised learning techniques. Ahmed et al. [11] provided a survey about network anomaly detection techniques, which focused on the few categories of detection techniques including few classification methods, statistical techniques with non-learning based techniques and few clustering approaches; moreover, the few dataset used for network anomaly detection was discussed as well, and the gap in this survey article was that it did not provide details about the pre-processing and feature extraction phases, which are very important in NADSs.

Although the available surveys in the literature have discussed the different properties of IDSs, most of the existing surveys have not covered ensemble learning approaches and neglected the pre-processing phase, which is an important task in network anomaly detection systems. The present survey provides a comprehensive discussion together with the fact that it conveys a better understanding of how anomaly detection and recognition systems are designed based on different types of machine learning techniques. A brief comparison of our survey with the existing survey articles is demonstrated in Table 1.

The main contributions of this survey are highlighted as follows:

- A systematic architecture for network anomaly detection and recognition systems is proposed from a user’s behavior point of view followed by the properties of intrusion detection systems and applications.
- The recent network data pre-processing tools for feature extraction comprising of feature creation, reduction, conversion and normalization are discussed.
- A comprehensive discussion on various shallow and deep learning techniques, such as supervised, unsupervised, new ensemble, and deep learning approaches are discussed followed by the challenges of designing an efficacious NADS.
- A detailed discussion of evaluation criteria, including evaluation metrics and several contemporary datasets applicable for NADSs, is provided.

### 1.2. Review Methodology

In order to perform this survey, journal articles and conference proceedings related to the IDSs, particularly machine learning-based anomaly detection systems as well as those which match the scope of this survey, were compiled. In the present survey, we followed a review direction according to the proposed conventional architecture of a machine learning-based network anomaly detection system which is depicted in Figure 1. A structured review methodology was applied in order to scrutinize the research studies on the main phases of machine learning-based anomaly detection systems as follows.

Firstly, this survey starts with a background of intrusion detection systems including the methods, properties, and multiple applications of anomaly detection systems in order to take a closer look at the fundamental concepts of this subject matter. In addition, contemporary malicious behaviors are described to simplify the meaning of abnormal behaviors in a massive amount of normal behaviors. Secondly, research articles are investigated from a pre-processing point of view to discuss how the features and network data were collected and extracted; this step helps to assess the quality of the pre-processing phase. Afterwards, as a critical module in anomaly detection systems, every research article was discussed from a machine learning perspective to categorize the research studies into four main groups of machine learning techniques which were supervised learning, unsupervised learning, deep learning, and ensemble learning techniques. Regarding the learning approach of every discussed article, we tried to discover their anomaly detection and/or classification methodologies to determine a summarized version of their learning logic. Moreover, we mentioned the gap and limitations together, accordingly proposing a future direction to address it. Meanwhile, the challenges for each category of machine learning techniques were highlighted at the end of the related sections. Furthermore, we reflected all the aspects and properties of anomaly detection techniques in the corresponding comparative tables, separately and in overall.

Fourthly, the evaluation criteria for network anomaly detection systems were discussed in detail to accurately evaluate the performance of anomaly detection systems. In this section, we discuss popular datasets which were applied in the area of anomaly detection fallowing by various evaluation metrics. Finally, some of the important issues with respect to different phases of NADSs were sorted into the main challenges and future directions of the proposed survey. We believe that this survey enabled numerous techniques of shallow and deep learning techniques to be synthesized, and enabled the strengths, weaknesses, challenges, and limitations of the previous studies to be identified.

The rest of the present article is designed in a way that, Section 2 puts into words the background of intrusion detection systems and properties. The data pre-processing and feature extraction are discussed in Section 3. Section 4 is dedicated to machine learning techniques for network malicious behavior detection and recognition. Section 5 argues various evaluation metrics and datasets used in NADS, and the challenges and future directions for the possible extension of NADSs and their applications are explained in Section 6. Finally, concluding remarks are proposed in Section 7.

## 2. The Background of Intrusion Detection Systems and Properties

An intrusion detection system (IDS) is typically a software application or device used to monitor network traffic for the detection of malicious activities or policy violations in network environments. These malicious activities are either reported to the security administrator of system or gathered centrally by the security information event management (SIEM) system. The monitoring environments fall into two categories: host and network-based environments [14]. A host-based IDS (HIDS) monitors the activities of a host via gathering information about various processes that occur in a computer system. Therefore, a sensor is deployed in the system to check the hosts and log the operating system activities, whereas a network-based IDS (NIDS) monitors network traffics to remotely identify anomalies spreading over a network connection [14,15].

### 2.1. Intrusion Detection Methods

Generally, intrusion detection techniques fall into four main categories: misuse-based detection system (MDS); anomaly-based detection system (ADS); stateful protocol analysis (SPA); and hybrid detection system (HDS) [16]. An MDS processes network traffics to compare the perceived input observations with attack signatures, any deviation from which can be considered as suspicious behavior. MDSs achieve a higher true positive rate (TPR) while facing known attacks, but fail to identify new or even the transfiguration of existing attacks. Increasing false alarm rates in detecting new attacks is an important issue from a security point of view, and is required to protect against these attacks. To address this drawback in MDS techniques, automatic signature generation (ASG) techniques have been proposed [17].

ADSs (also called behavior-based detection system) first establish a normal profile and create a performance baseline under normal operating conditions. The detection system continuously monitors all input traffic and simultaneously compares against the predefined baseline, and any observation outside the baseline considered as an abnormal (malicious) behavior will send an alert to the security administration system [14]. Unlike MDSs and ADSs, which rely on host or network-specific characteristics, stateful protocol analysis (SPA) depend on vendor-developed universal profiles that determine how specific protocols are utilized. HDS is a collaborative detection system that employs multiple network characteristics and observations, such as user behavioral profiles, attack signatures, and stateful protocols [18,19].

### 2.2. Applications and Deployments

Generally, the deployment architecture of an IDS has two categories: centralized and distributed. Centralized IDSs are usually deployed at only one edge of a system with a non-compound architecture. This type of installation tool relies on the size of the system and the sensitivity of the network data. A distributed architecture is a composted system which contains multiple subsystems of IDSs. This type of deployment assists in the detection of malicious instances, and can recognize relevant attacks from several locations [13]. IDSs are installed in various applications and systems such as Cloud computing (installed over nodes of centralized networks), Internet of Things (IoT) (installed to protect various applications and objects connected to the Internet), Mobile edge/Fog computing (installed close to networks edges to defend private data that transmit over mobile), and data center-based IDSs (installed on key servers over data centers and contains a set of storage and networked computers).

A Cloud-based IDS is required to firm migrated Cloud services to public Cloud environments. For example, Microsoft Azure and Amazon Web Services protect the infrastructures, software, and platforms [20]. Existing NIDSs fail to identify and respond to internal malicious behaviors; nor they are capable of protecting Cloud-based infrastructures. Therefore, a scalable and collaborative IDS is required to be installed in the Cloud to monitor normal and malicious instances [21]. In the case of IoT-based IDS, the existing NIDSs used in IoT are not sufficiently capable of handling a huge number of alerts with high FARs, because of the overlap between normal and malicious observations [21,22,23]. Autonomic NIDSs containing a self-paradigm are essential in IoT applications and the new generation of NIDSs should be systematically improved with low human interventions.

In Mobile edge/Fog computing, one of the main issues is monitoring the traffic connection between Cloud and Edge environments, as these technologies demand scalable NIDS techniques that can effectively identify malicious observation in real time. Installing IDSs over the edges of networks can help to overcome Cloud challenges to process large-scale networks with high mobility and low latency. The main challenge of Edge computing compared to the Cloud is its distributed architecture. Edge computing has a distributed norm, whereas the Cloud paradigm has a centralized norm. The issue of the distributed norm in Edge computing is the integration of various service infrastructures [13]. The task of data center-based IDS is the investigation of all network data that are transmitted between clients and servers and vice versa. Data center-based IDSs should provide a solidified security system and monitor malicious observation over the devices and servers.

As a result of all these factors, machine learning based-IDSs deployed over the mentioned applications should be capable to detect all types of contemporary malicious instances discussed in Section 2.3, including traditional known attacks and new zero-day attacks. Such IDSs are expected to effectively and efficiently handle high-speed network traffic data and transmit network data at 10 Gbps or higher. In addition, these services should be self-adaptive and scalable to process various networks through large areas in real time [13].

### 2.3. Contemporary Malicious Behaviors (Network Attacks)

The quantity and variety of network attacks are dramatically growing, causing financial losses, interrupting businesses, and stealing users’ confidential information. Referring to the Australian Cyber Security Center (ACSC) [24] and McAfee threat reports [25], contemporary attacks still expose network and computer systems and require further improvement using NADSs. The different types of malicious behaviors are explained as follows:

- **Trojan** is a malware often disguised as normal software but carrying out abnormal activities in the backend. Trojan malware are usually utilized by cybercriminals to penetrate victims’ systems and the users are mostly cheated by hackers to execute the Trojan malware on target computers [26].
- **Scareware** is a new sort of malware created to deceive users to buy and download useless and potentially dangerous software—for instance, fake protection programs that cause many financial and security-related perils to the users [3].
- **Rootkits** are malware created to hack particular processes and enable continued privileged access to victims’ machines. Rootkit malware can be executed at various levels such as application programming interface (API) calls, at the user level or interfere with OS at the device level [27].
- **Analysis** contains different types of port scanner attacks such as spam and .html file penetrations [14].
- **Ransomware attack** is a malicious software from cryptovirology that threatens to reveal the victim’s sensitive information unless a ransom is paid [3].
- **Zero-day attack** is a computer-software vulnerability that exploits a significant security infirmity without the creator’s awareness. Until the vulnerability is identified by the system, hackers can exploit it to negatively affect the computer programs and data [13].
- **A Botnet** occurs when the number of hijacked systems remotely controlled via malware operators perform malicious activities. Cybercriminals infiltrate targeted devices by using typical Trojan viruses to penetrate the computers’ security systems. Some example of these malicious activities are DDoS attacks, credential-stuffing attacks and Web application attacks [13].
- **Brute force** happens when an attacker submits massive pairs of username and passwords or passphrases with the hope of finally guessing correctly. The attacker systematically checks all possible passwords to find the correct one [28].
- **Backdoors** are the techniques that attacker uses to remotely gain access and control of the victim’s computer by normally responding to client applications. Many of these techniques utilize the IRC backbone and often obtain commands from IRC users’ chats [14].
- **Denial-of-service (DoS)** is a malicious activity which tries to make a computer, OS or server unavailable to its client by temporarily or permanently interrupting the host services connected to the Internet [2].
- **Exploits** can be represented as a series of comments or a peace of code that often enable cybercriminals to discover a security-related issue in an OS to remotely control the whole machine [14].
- **Distributed denial-of-service (DDoS)** happens when multiple computers flood the bandwidth or resources of a targeted system, generally one or more web servers [29].
- **Fuzzers** is a black box malicious software testing technique that tries to create a program or network to interrupt the target machine by injecting randomly generated data such as numbers, chars, metadata or pure binary sequence [14].
- **Generic** is initially a collision attempt on the private keys of ciphers. For example, if a cipher takes an N bit key, in response, the generic attack gets a ciphertext and tries to decrypt the original cipher with all possible $2^{N}$ keys [14].
- **Reconnaissance** is a kind of data collection from networks or other services trough illegal ways. In this sort of attack, cybercriminals try to obtain information about the victim’s network or computer to use in an unauthorized investigation [29].
- **Shellcode** is basically the payload of another attack. The malicious orientation provides a command-line to the attacker in order to give access to a computer, all with the benefits of the procedure being abused. When an exploit builds up a connection to the vulnerable procedure which is not already closed, the shellcode can later re-utilize this connection to negotiate with the attacker [14].
- **Shellshock attacks** usually breach the command-line shell of OSs such as, Apple, Linux, and UNIX. The attacks were discovered in 2014 and at the time, many computer systems had been penetrated by a remote code execution which had achieved full access and control [3].
- **Worms** duplicate their own code in order to spread it to other computers. These often employ a network environment to propagate themselves and occur when there are security failures on the target computers [14].

## 3. Data Pre-Processing and Feature Extraction

Data pre-processing is a significant step in every machine learning technique and may take a considerable amount of time in the whole anomaly detection process. This step involves feature creation, reduction, conversion, and normalization processes in order to find the most informative features. Then, the desired feature set will be fed—as input parameters—to the learning system module. In addition to informative data, the extracted network data also contain unnecessary and duplicated information, which negatively affect the detection performance as well as time. Pre-processing refines the network-related data and removes unnecessary and noisy instances; thereafter, the extracted features will be suitable inputs for the detection phase. In NADSs, the main steps of the pre-processing phase include feature creation, feature reduction, feature transformation, and feature normalization as explained below [14].

### 3.1. Feature Creation

**Figure 2:**
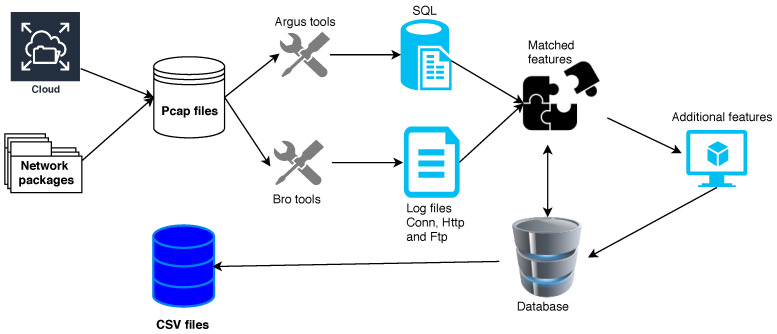
The flow process of feature generation from Pcap files to CSV files.

Initially, network traffic features are extracted using various collection tools, such as Netmate, BRO-IDS, Argus, Netflow and Tcptrace. In anomaly detection systems, it is essential to accurately characterize the potential features of every user to detect all malicious activities. Therefore, different features like the extracted features in the UNSW-NB15 and the KDD99 datasets (flow features, basic features, content features, time features, and generated features) should be meaningfully and accurately extracted. Moreover, in these two datasets, additional features are constructed using both transactional connection times and transactional flow identifiers to mathematically represent the potential attributes of network observations [30]. The flow process of feature generation from Pcap files to CSV files are depicted in Figure 2. In Table 2, the different categories of network features in the UNSW-NB15 dataset and the corresponding feature numbers are listed. More details about the UNSW-NB15 and KDD99 datasets are explained in the Section 6.

### 3.2. Feature Reduction

Feature reduction step plays a crucial role in network data pre-processing that filters and removes noisy and useless instances. In a real-time raw network traffic collection scenario (e.g., Tcpdump and Libpcap), the number of normal features are usually significantly greater than the malicious ones. Hence, such unbalanced datasets usually face a huge number of duplicated and redundant features, which are not required to reduce the malicious detection performance. On the other hand, it is required to extract the potential and informative features, which contain important information. Meanwhile, these features should be cautiously analyzed to segregate only appropriated data, which assists in the detection phase to accurately identify malicious instances [14].

Independent component analysis (ICA) [31], principal component analysis (PCA) [32] and association rule mining (ARM) [20], are popular techniques for selecting and reducing important network data features [33,34,35]. PCA is a famous linear feature reduction method that requires less memory storage and less processing time compared to other mentioned techniques [36].

The feature selection component consists of four steps, subset generation (constructed from original feature set), subset evaluation, stopping criterion, and the final selected feature set [1], which are explained below:

- **Subset generation:** is an informed (heuristic) search strategy that generates subset candidates from the original search space. For instance, in a dataset including *f* features, there are $2^{f}$ possible subsets of features.
- **Subset evaluation:** every generated subset needs to be evaluated based on appropriated metrics. These metrics can be applied to the selected subsets based on learning techniques, dependently or independently [14].
- **Stopping criterion:** is a conditional step to finish the feature selection process, with common rules, such as the maximum number of iterations or the minimum selected features.
- **Result validation:** in this step, the result can be evaluated based on estimating the output of reduced features using a priori information.

### 3.3. Feature Conversion

Network traffic data consist of different parameters, such as time, flow, content, and basic features, and every feature includes various properties in the original version of the dataset. These features will be finally represented in multiple qualitative (i.e., symbolic) and/or quantitative (i.e., numeric) data categories. Since most detection techniques can only handle the integer data type, the features that are not in the integer type, must be converted into a unified numeric format [13].

### 3.4. Feature Normalization

In this step, the feature values need to be normalized and scaled down/up into a suitable interval. The main advantage of normalization is removing the bias from the raw network instances without losing statistical properties. In order to accomplish this process, techniques such as Min–Max (a linear transformation function [13]) can be used for the normalization of features, as given in Equation (1):

(1) $$\begin{array}{c} X_{normalised}=\frac{\left(X-min(X)\right)}{\left(max(X)-min(X)\right)}\; \end{array}$$

In Equation (1), *X* defines the value of the feature, *min*(*X*) represent the minimum value of the feature and *max*(*X*) represent the maximum value of the feature among all values in a pattern.

Jamdagni et al. [37] proposed a technique to analyze and construct features from network traffic using Wireshark. The authors used n-Gram text categorization to extract raw network features and transformed into feature vectors. Every packet payload was represented by a feature vector in a 256-dimensional feature space based on the following formula:

(2) $$f_{i}=\frac{O_{i}}{\sum_{j=1}^{256}O_{j}}$$

where $O_{i}$ is the occurrence of *i*th n-gram. The overall value of the relative frequencies is given by $\sum_{j=1}^{256}f_{i}=1$ [37].

## 4. Machine Learning Techniques for Network Malicious Behavior Detection and Recognition

**Figure 3:**
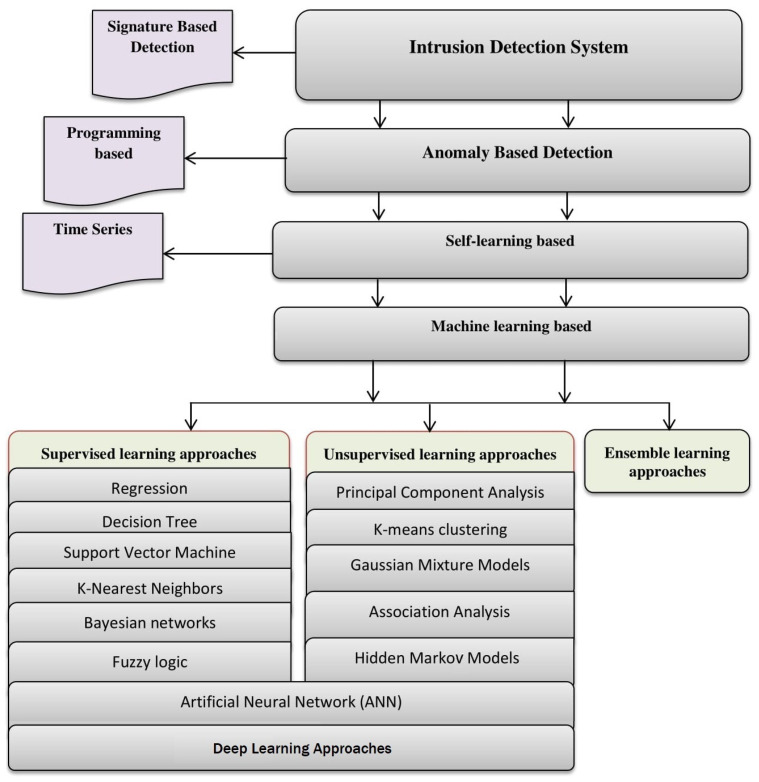
Classification of intrusion detection systems.

The detection and recognition phase of a network anomaly detection system (NADS) is an essential component to accurately discover malicious activities. Basically, machine learning-based NADSs approaches are classified into four main categories: supervised leaning, unsupervised leaning, deep leaning, and ensemble learning approaches, which are illustrated in Figure 3, and described as follows:

### 4.1. Supervised Learning Approaches

#### 4.1.1. Regression Techniques

- **Regression:** is a set of statistical processes that specify the relationship between one dependent value and one or more than one independent values. This method is divided into two main regression techniques, linear, and polynomial [38].
- **Decision Tree (DT) learning:** is a classifier technique based on tree structure. Every node in this structure is correlated to a specific feature in the dataset and the cost (weight) of the connected edges are feature values. Each leaf node of the tree structure represents a malicious behavior in a DT-based detection system [39]. Generally, decision trees fall into two main categories: regression tree analysis and classification tree analysis. Regression tree analysis is when the predicted outcome can be considered a real number, whereas classification tree analysis is when the predicted outcome is the class (discrete) to which the data belong.
  Narouei et al. [40] proposed a malware detection technique based on structural mining. The behavioral features have were extracted from a dynamic-link library dependency tree. In the detection phase, similar measurements such as Cosine (M1), Jaccard (M2), Pearson (M3) were used to detect the similar variants or to obfuscate the versions of a malware, so the drawback of this paper is the identification of only Malware and benign programs with the known tree’s string encoding format.
  Singh et al. [41] proposed a random forest-based decision tree technique. The authors reported that the random forest method utilized for Botnet detection achieves a high accuracy of prediction. This technique is also capable of processing various bots and further characterizes the data by a large number of multiple types of descriptors, though only peer-to-peer (P2P)-based Botnet anomalies were studied in this research work, which could be a limitation in new computing technologies. Jabbar et al. [42] designed an ensemble classifier scheme based on two learning algorithms, namely random forest and average one-dependence estimator (AODE). The AODE overcomes the feature dependency problem in naïve Bayes classifier and the RF technique enhances the overall accuracy while it reduces the misdetection rates, while one of the main limitations of the proposed technique is the necessity of labeling the attack data patterns.

#### 4.1.2. Classification Techniques

**Figure 4:**
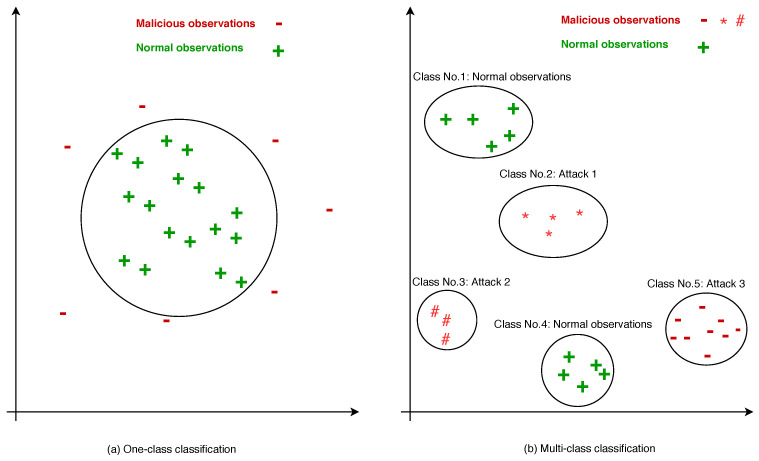
Illustrated example of one-class classification (**a**); and multi-class classification (**b**).

Classification is a technique of assorting behavioral instances into predefined classes over a network dataset. The training dataset normally contains approximately 75% of the whole dataset while testing the dataset contains the remaining instances for evaluation purposes. Classification based-techniques are categorized into two types: linear and non-linear classification. In the case of network data, the network observations are basically labeled ‘0’ and ‘1’ (such as 0 for malicious observation and 1 for normal) as depicted in Figure 4a for one-class classification and in Figure 4b for multi-class classification.

In real-time network traffic, the normal observations are considerably greater than malicious observations (attacks), which is the nature of the network traffic data. In this scenario, linear anomaly detection methods or one-class anomaly detection are the most popular and appropriated techniques. Conversely, multi-class techniques are useful when the normal and malicious observations are equally existent in the dataset in order to classify different types of attacks.

The following methods are the most frequently used classification techniques in network anomaly detection and recognition systems.

- **Classification tree:** is a test design technique which is used in various areas of software development. The classification tree methodology includes two main phases: identification and combination. The first phase is the identification of test-relevant aspects, called classifications in pattern recognition domains, and their corresponding values, called classes. The second phase is the combination of different classes from all classifications into test cases.
- **Support vector machine (SVM):** is a successful maximum margin linear classifier system. A typical SVM classifier contains two main steps to classify network data instances.
  Firstly, the training dataset is transferred to a higher dimensional feature space and then, using a kernel function, the linear non-separable problem converts into a linearly dividable problem. In anomaly detection problems, all normal instances are placed in one class and different malicious land in another class. Afterwards, the network observations are over a hyperplane with the highest margins at the closest spots on every sector. Only the patterns that are very close to the margin, affect the computation of these margins. The remaining patterns could be eliminated without influencing the final results.
  Ambusaidi et al. [43] proposed an IDS named the least square support vector machine (LSSVM-IDS), which has the capability to handle both linear and non-linearly dependent data instances. By using LS-SVM, the network attacks are classified into DoS, Probe, R2L, and U2L. Moreover, the performance classification for these attacks evaluated based on three datasets (KDD CUP 99, Kyoto 2006, NSL-KDD), labeling the patterns to train the detection system, is one of the main challenging gaps in this study.
  Kang et al. [34] described a one-class classification method to enhance the performance of intrusion detection for harmful attacks. The outcome results was evaluated based on artificially generated instances in a two dimensional space. In the detection phase, the authors followed a simple logic, the center of the normal patterns was located at (0, 0), and two malicious class centers were at (1, 1) and (−1, −1), respectively. Experimental results over simulated data show better performance and then extracted data in the DARPA dataset. Perdisci et al. [35] presented several one-class SVM methods for ADSs based on the Harden Payload. The authors constructed several SVM classifiers and each classifier was applied on a various observation from the payload. The experimental analyses showed that the combination of the obtained classifiers improves both the detection accuracy and the hardness of evasion, the main gap of this study is the complexity of feature selection and labeling process of the features. Some other works for malicious detection using a one-class support vector machine are reported in [44,45,46,47].
- **K-nearest neighbors (KNN):** is a supervised machine learning technique that uses a pattern and classifies new neighborhood patterns by considering a similar measure based on various distance functions. In malicious detection applications, the classification is performed by a majority vote for its nearest neighbors to the object and classifying malware, while if it is benign then it is based on the closest training instants in the Windows API calls [48].
  Alazab [48] proposed a profile-based classification technique to analyze the behavior of malicious observations based on KNN. The authors statically and dynamically extracted various features from the malware to represent the behavioral type of its code, such as Windows Application Programming Interface calls. KNN techniques have been used to profile malware behaviors and to categorize them into malicious and normal classes [48]. One of the main gaps in this paper is only using API calls to reflect the behavior of user, therefore, it would be very hard to identify contemporary malware. KNN-based NADSs first establish a normal network profile, and like other binary classification problems, any deviation from the normal profile is considered malicious. KNN is a strong anomaly detection technique since adapting parameters is not required in the training phase. Nevertheless, these techniques are mostly time-consuming and need a large storage space for the classification of high-speed traffic data, though some other similar works can be found in [49,50].
- **Bayesian networks (BNs):** are graphical models, with evidence propagation controlled by the Bayesian theorem. BNs are inherently sturdy for missing information, and are better adapted to categorical information compared to distance-based classifiers [51]. The structure of BNs and its representation is understandable for human operators, in comparison with other machine learning approaches. This structure allows modeling the flow of information through the network and traces the causes of malicious instances [51].
  Xu et al. [52] designed an intrusion detection model by proposing a hierarchical continuous time Bayesian network (CTBN). The system traces the network packets and applies the Rao-Blackwellized bit filtering to learn the parameters, the combination of system calls and network events in feature selection and extraction phase provided strong abilities to identify anomalies. Altwaijry [53] developed a Bayesian-based intrusion detection system to detect anomaly activities over network traffic. First, the different sub-attacks were detected, then they were classified into four main attacks: including DOS, Probe, R2L and U2R. Initially, the system was developed to recognize intrusions by using a naïve Bayesian classifier; eventually, the technique extended to a multi-layer Bayesian-based intrusion detection.
  Moustafa et al. [54] proposed a malicious behavior classification approach using a correntropy-variation technique. The authors believe that modern network attacks can mimic normal activities and make it very complicated for the network to trace the malicious observations. The authors designed a network forensic technique for investigating network-based attacks. In the first step, network traffic data were captured; afterwards, the authors extracted significant features using the chi-square statistic. Finally, malicious instances were detected by applying the correntropy-variation technique, and the proposed statistical technique that the authors proposed was not a learning-based technique as well as not applicable to Cloud and Fog computing environments—which are gaps we identified in this research study. [54]. Another research study into anomaly-based IDS using kernel density estimation can be found in [55].
- **Fuzzy logic (FL):** FL has been applied in IDSs for two main concerns [56]. Firstly, there are plenty of quantitative network parameters related to network packets and other environmental properties. The parameters involved in the network anomaly detection systems, such as CPU usage time, protocol type, connection interval, etc., could potentially be represented as fuzzy variables and equivalent rules. Secondly, as described by Bridges et al. [56], the concept of security itself is fuzzy. That is to say, fuzzy rules smooth out the unexpected deviation of normal instances against the malicious ones [56].
  Dickerson et al. [57] introduced the fuzzy intrusion recognition engine (FIRE) by applying the fuzzy rules and fuzzy sets. This technique employed different data mining methods for processing network packages and created fuzzy sets for network data instances. Later, the fuzzy sets were applied to make fuzzy rules for detecting malicious instances against normal ones, [58], and the main gap in this research work was that the feature extraction was not properly done in the pre-processing phase.
  Haider et al. [59] provided a dataset used for intrusion detection by trying fuzzy qualitative modeling. The issues of realistic assessment and systematic metric for evaluating different types of IDS datasets were investigated. Practically, it is difficult to access and obtain real-world network traffic data due to business stability and integrity issues. In order to achieve this, in the first step, the authors established a metric based on fuzzy logic to assess the quality of the realism of existing intrusion detection datasets. Afterwords, a synthetically realistic intrusion detection dataset was developed using the presented metric results. In this study, only DoS and DDoS attacks were investigated, which is the main limitation of the proposed detection system.

#### 4.1.3. Challenges and Future Directions for Supervised Learning Techniques

The main assumption in supervised learning approaches is the availability of an appropriate training dataset. In addition to this, the dataset should contain labeled data for every class, including normal and malicious instances. Every known/unknown input datum goes through a comparison against a predefined model in order for the belonging classes to be determined. Generally, there are two major drawbacks for applying supervised learning techniques that the researchers in the area of network anomaly detection may consider for future research studies. Firstly, malicious instances are always significantly less numerous than normal observations in any training dataset. Therefore, this problem causes an imbalanced class distribution in the dataset during training. Secondly, preparing accurate and characterized representative labels, particularly for the instances in the malicious class, is usually a challenging task. Some techniques inject artificial malicious patterns in a normal dataset to collect a labeled training dataset [14].

### 4.2. Unsupervised Learning Approaches

#### 4.2.1. Clustering Techniques

In anomaly detection, clustering is the manner of grouping a set of network data instances in such a way that all malicious instances in the same batch (called a cluster) achieve higher similarity as compared to the other groups (clusters which can be normal instances). Another method that is technically close to the clustering concept is that of outliers, which define some data instances as more highly digressed than the usual groups in a data space.

**Figure 5:**
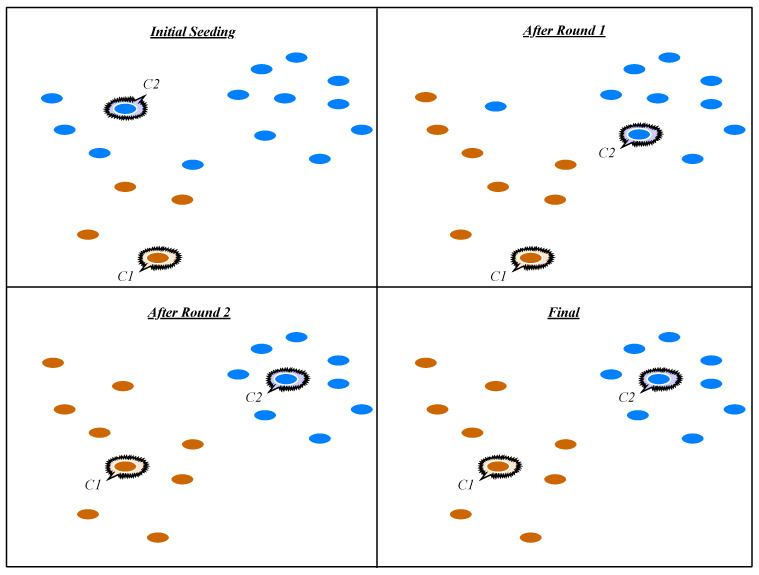
Pictorial representation of K-means clustering.

Clustering-based network anomaly detection techniques have several advantages as compared to classification techniques. Firstly, the network data are classified in an unsupervised fashion and do not require class labels for all the network features. Secondly, clustering techniques are effective in large datasets, by dint of the fact that they reduce computational complexity and achieve better performance than the other classification techniques. Hence, the first disadvantage of clustering-based network anomaly detection would be the fact that these techniques are highly related to the efficacy of creating a profile for normal instances. Secondly, clustering is often time-consuming for dynamically updating a profile for legitimate network instances:

- **Hierarchical clustering:** is a cluster analysis method that attempts to generate a hierarchy of clusters and technically falls into two main categories: agglomerative clustering, which is a bottom–up modeling approach, and the other one, divisive, is top–down clustering. Horng et al. [60] introduced an SVM-IDS based on hierarchical clustering. The clustering method provided a classifier with higher-qualified training patterns which were extracted from the training dataset, and one of the limitations of this technique was that despite its high performance in two attacks in the KDD Cup 1999 dataset (DoS and Probe), it was not acceptable for the U2R and R2L attacks.
- **K-means clustering:** is a famous unsupervised learning approach applied to clustering problems. The technique follows a simple strategy to classify a given number of patterns in a dataset through a predefined number of clusters. A standard type of K-means algorithm (naïve K-means) can be created by repeating two steps. The first step is assigning each observation to the cluster whose mean has the least squared Euclidean distance. The second step is updating and calculating the new means (centroids) of the observations in the new clusters [61]. Figure 5 shows a pictorial representation of K-means clustering.
  Lee et al. [62] proposed a proactive detection technique for DDoS attacks using cluster analyses. The cluster algorithm that the authors used, contains two main types of clustering algorithms; hierarchical clustering and partitioning clustering, to pre-determine the number of clusters, the limitation of this work is the weakness of the system to identify other attacks such as R2L, U2R and probing, with the exception of DoS and DDoS attacks. Similarly, Li [61] implemented an anomaly detection model based on clustering analysis using the K-mean clustering algorithm. In this research, the author reported some limitations, such as the sensitivity of the algorithm to initial conditions, outliers, and noise. Even if an object is quite far away from the centroid of the cluster, it is still directed into a cluster. Thus, it re-curves the cluster shapes.
  Costa et al. [63] designed an optimum-path forest clustering technique to estimate the probability density function (pdf) employed in clustering algorithms. The authors applied this clustering technique for intrusion detection systems by speeding up the optimum-path in forest clustering. The feature selection and extraction process is not clearly explained in this study and this can be the main gap of this research work. Jadhav et al. [64] proposed a scheme for a network anomaly detection system based on packet signature clustering and network analysis. The clustering technique follows a simple rule; whenever the input network instances match one of the intrusion signatures, the system reacts to the security administrator concerning the possible threat in details, one of the limitations of proposed system is the paucity of extracted features, which is a lot less compared to the features in popular datasets such as UNSW-NB15, KDD99, and NSL-KDD datasets.
  Although various clustering techniques have been used for NADSs, the most utilized techniques for malicious detection are regular and co-clustering techniques using different strategies and processing methods [9,65,66]. For example, the K-means, as a regular clustering method, assembles features from the dataset instances, but co-clustering techniques concurrently consider both features and instances in the dataset to make clusters.
- **Gaussian Mixture Models (GMM):** is a technique of probabilistically representing normally distributed sub-sets throughout a dataset. A finite mixture mechanism is presented as a convex collection of two or more PDFs, and the joint attributes of these PDF functions can predict any random distribution [67].
  Moustafa et al. [67] introduced an outlier Gaussian mixture (OGM) model for discovering zero-day attacks based on the concept of network abnormal behaviors. The authors collected the Web application data and extracted the related features. After preparing significant features, the normal model was created by using the outlier Gaussian mixture (OGM) technique based on GMM. Some other techniques for anomaly detection using GMM have been reported in [20,68,69,70].

#### 4.2.2. Dimensionality Reduction Techniques

These techniques basically try to obtain a set of principal variables by diminishing the number of random variables under statistical consideration [71]. The popular dimensionality reduction approaches used for anomaly detection systems are described in the following:

- **Principal component analysis (PCA):** is the main linear technique and a well known unsupervised dimensionality reduction method, which determines the principal directions of the data distribution. In order to acquire these principal directions, one needs to create the co-variance matrix of data and calculate its conquered eigenvectors [72].
  Han et al. [73] developed a naïve Bayesian NADS based on PCA. The system calculates the attribute value of the original network dataset, then extracts the essential properties using PCA. With the aim of improving the traditional naïve Bayesian classification method, the authors took the main properties as the new features and the corresponding principal component contribution rate as weights, though a drawback of the proposed technique is the complexity of labeling attack data. Bhagoji et al. [74] introduced a dimensionality reduction method to protect the networks against evasion attacks on machine learning classification techniques. The authors incorporated PCA to increase the flexibility of machine learning techniques, targeting both the classification and the training phases, though there are two limitations in this work, insufficient on its own and a lack of universality, where it falls short of being a comprehensive defense mechanism against evasion attacks, the authors suggested using other dimensionality reduction techniques for addressing these limitations. Ding et al. [75] proposed a PCA subspace model for anomaly detection in high-dimensional data space. The authors introduced a model of compressed PCA subspace projection and characterized key theoretical quantities, relating to its usage as a tool in malicious detection. In addition, the technique and application implemented for identifying IP-level volume anomalies in network traffics, the important phase of an IDS which is pre-processing and feature extraction was not covered in this paper and the evaluation criteria to assess the technique is missing.
- **Independent component analysis (ICA):** obtains the independent variables by maximizing the statistical independence of the estimated components. As an example for this technique in network anomaly detection, Palmieri et al. [31] proposed an approach based on ICA. The authors created a two-phase anomaly detection scheme using various distributed sensors located in the entire network. By using this dimensionality reduction technique, the authors modeled the anomaly detection as a blind source separation problem, and the gap identified in this research work is that analyzing fundamental independent traffic is time-consuming and predicts a weak performance in high-speed network traffic data.

#### 4.2.3. Association Analyses Techniques (Hidden Markov Models (HMMs))

HMMs are generative models employed to characterize stochastic procedures. HMMs are appropriated methods for modeling the dynamic behaviors of underlying systems, and these models are popular in the pattern recognition area. HMMs are often applied to construct time series models and also successfully used on different domains of network anomaly detection systems. Moustafa et al. [21] proposed a threat intelligence scheme based on mixture hidden Markov models (MHMM). The MHMM technique is utilized by the Gaussian mixture model (GMM), whenever the quantity of mixture components is known, and borders of perceived data are unlimited (i.e., (−∞,+∞), and a disadvantage of the proposed system requires a large number of normal and attack instances to accurately estimate the BMM and HMM parameters, moreover, the system also needs a new functions that enables running the algorithm for adjusting the sliding window to be implemented [21]. Some other similar works for malware detection using HMMs have been conducted in [76,77].

#### 4.2.4. Artificial Neural Network (ANN) Techniques

ANN or connectionist systems are inspired by biological human brains. Traditionally, ANN-based anomaly detection techniques have been used for host-based IDSs that focus on predicting any divergence from the normal profile as a sign of an abnormality. In the ANN-based IDSs, the network has the ability to learn in the training phase and predict the behavior of different users. The main superiorities of ANN techniques are firstly its tolerance to the uncertain or the wrong data and secondly its capability to predict the anticipated outputs without having previous knowledge and predefined labeling of the input data. These properties are what made ANN an appropriated approach to NADSs; however, there are several drawbacks in neural network-based techniques. The first one is that ANN-based approaches may not achieve acceptable results due to weak learning function or insufficient data. Second, the training phase of ANN is often slow due to data feeding and also adjusts the weights for all neurons while back-propagating the errors.

Saber et al. [78] proposed an IDS based on ANN. The purpose of the work was to design an optimized neural network with crucial parameters for anomaly detection which was capable of detecting different kinds of attacks. In the first phase, basic attributes were extracted to nourish the input layer and to verify the dependence between these parameters and malicious instances. In the second phase, the authors incorporated the parameters according to content in order to prove the efficacy and also to show in what situation the parameters are crucial; their proposed technique works well for identifying three attacks in the KDD datasets (Probe, DOS and R2L), however, it detects U2R attacks with a low detection rate.

Rabbani et al. [14] proposed a hybrid machine learning approach based on a probabilistic neural network for malicious behavior detection and recognition in Cloud computing. The authors designed a particle swarm optimization-based probabilistic neural network (PSO-PNN) to firstly detect the malicious instances against normal ones and then recognize the type of abnormality based on an attack classification algorithm. In addition, the UNSW-NB15 datasets were exploited to assess the malicious detection and recognition techniques, though one of the limitations of this study using the PSO-PNN technique is finding high intra-class similarity in the backdoors attack and DoS attack observations. Ramadas et al. [79] proposed a technique named anomalous network-traffic detection with a self organizing map. The technique attempts to establish a two dimensional SOM for every monitored network, and then the neural network was trained using normal traffic instances in the training phase to learn the network properties. This technique has been tested using the DNS and HTTP services, and the limit of this research work is that the infrequently occurring corner-case behavior might be identified as malicious. Some other ANN-based anomaly detection approaches have been conducted in [80,81,82,83,84,85].

#### 4.2.5. Genetic Algorithm (GA) Techniques

In search-based problems, GA is used to find approximate solutions to optimization. In IDSs. this technique has also been widely used to discriminate malicious activities against normal ones in network traffic instances. The main superiority of GA in malicious detection systems is its robustness and flexibility as a global search method. Moreover, the GA search strategy relies upon probabilistic search rules in lieu of deterministic rules [58]. In the area of NADSs, GAs have been applied in numerous ways. Folino et al. [86] proposed a distributed anomaly detection scheme based on ensemble GA. Network instances distributed on various independent websites and the trainer system extract useful information from these data. Then, a normal profile was used to identify anomaly instances. The experiments were assessed on the KDD Cup 1999 dataset, meanwhile the author did not provide information about how the features were selected and extracted in the KDD Cup 1999 dataset. Pillai et al. [87] introduced a network anomaly detection system based on GA. In the first step, the network sniffer traffic was analyzed to create a dataset, and in the next step, the rule set was automatically generated. Using the generated rule set and GA, the authors implemented a network IDS to analyze the particular portions of a network and prevent the traffic overflow. Another similar work based on GA was proposed, though the gap identified in this research work was the fewer number of features that they considered in the feature selection phase.

#### 4.2.6. Challenges and Future Directions for Unsupervised Learning Techniques

Training data are not necessary in unsupervised learning techniques, and this can be a great advantage and appropriate technique which have been widely utilized in the literature. Unsupervised learning techniques assume that legitimate observations are far more frequent than malicious instances in the testing dataset. Whenever this expectation does not occur, these techniques face high FAR. In this scenario, semi-supervised learning techniques are applicable in an unsupervised fashion with an unlabeled portion in the training dataset. Semi-supervised learning techniques assume that the network data in the testing dataset include signficantly less malicious patterns and the model learnt during the training process is solid against those few malicious instances.

### 4.3. Deep Learning-Based Anomaly Detection Techniques

**Figure 6:**
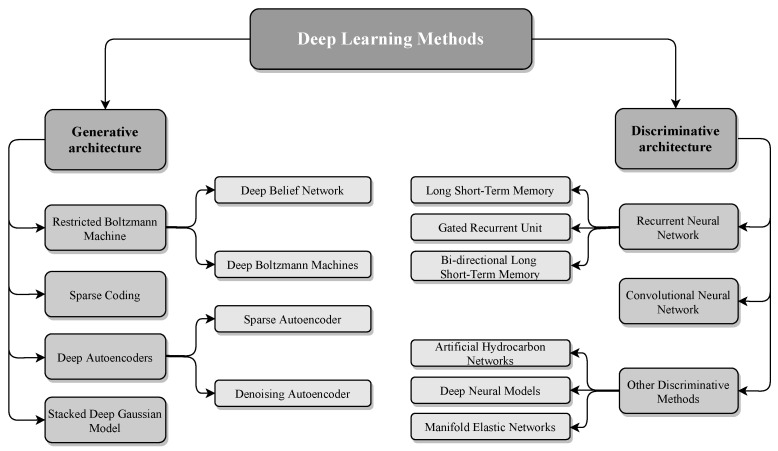
Taxonomy of recent deep learning methods.

Deep learning is a subdivision of machine learning in AI with the capability of learning in an unsupervised fashion from unstructured and unlabeled patterns. The foundation strategy behind deep learning in comparison with traditional ANN techniques is the employment of an advanced neural network in both the training and feature extraction processes. In shallow learning ANN, the network consists of one or two hidden layer(s). On the other hand, the network structure of deep learning consists of several hidden layers with various architectures [78]. Recently, deep learning techniques have become very popular in the area of pattern recognition and network applications. This is due to their intellectual properties such as fast learning in an unsupervised fashion, a quick and in depth computational process, and handling massive amounts of data. In NADSs, both traditional and deep learning networks need basic information about the normal traffic data to systematically design an appropriate structure. This network contains convenient middle layers to train the weights of the network and establish a model that can distinguish malicious instances against normal ones [88,89]. The architecture of deep learning networks is mainly divided into two models—discriminative and generative—as illustrated in Figure 6 and detailed in the following.

#### 4.3.1. Discriminative Deep Architectures

**Figure 7:**
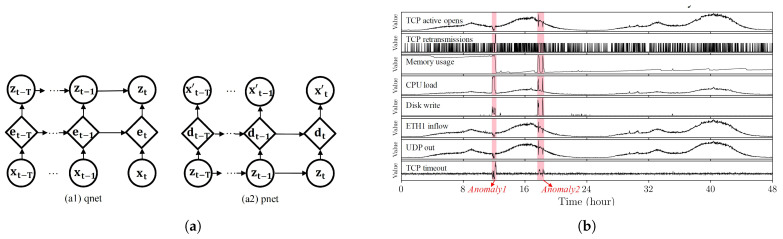
(**a**) A graphical representation of an RNN-based anomaly detection; (**b**) an example of a multivariate time series snippet with two detected malicious regions are highlighted in red.

- **The recurrent neural network (RNN)** is a model of deep learning techniques where connections between nodes (neurons) form a directed graph along with a temporal sequence and allows the network to expose temporal dynamic behavior. In this architecture of the neural network, the input data are linked in long sequences via a layer-by-layer connection with a feedback loop [89].
  Based on the connection between layers, RNN consists of two types: Elman and Jordan. The Elman type includes three main layers (input, hidden, and output) and also one context layer. The hidden layer has a connection to the context layer and after every feed-forward training progress, a copy of the previously hidden units are stored at the nodes of context layer. Jordan networks perform similar to Elman but the context nodes are directly fed from the nodes in the output layer [89].
  Bontemps et al. [90] proposed an anomaly detection model based on long short-term memory recurrent neural network (LSTM RNN). In this technique, the LSTM RNN is firstly employed as a time series anomaly detection model and the prediction of a current observation depends on both the current and its previous observations. Secondly, the technique was adapted to detect aggregate malicious patterns by generating a circular array. The circular array includes previous prediction errors which were stored from a specified number of the latest time steps. If the prediction errors were greater than the predefined threshold and lasted for certain time steps, these will be detected as anomalies. The model was created on a time series version of the KDD 1999 dataset.
  Shone et al. [91] proposed a deep neural network(DNN) that consisted of 100 hidden units. The model incorporated the rectified linear unit activation function and the ADAM optimizer for network anomaly detection. This technique was implemented on a GPU using TensorFlow, and the performance of the method was evaluated using the KDD99 dataset with the average accuracy rate of 99. Moreover, for any future works, the authors suggest the improvement of the method by using RNN and long short-term memory (LSTM) models [91]. Maya et al. [92] proposed an RNN model based on delayed long short-term memory (dLSTM) for network malicious pattern detection on the time-series data. In the first step, a predictive model was generated from normal traffic instances, then identified malicious patterns based on the prediction error for observed data. To deal with the various states in the waveforms of normal traffic data, which reduces the prediction accuracy, the authors applied multiple prediction techniques based on LSTM for malicious detection, pre-processing phase, feature selection and evaluation criteria are missing gaps in this survey.
  An overall graphical RNN model is represented in Figure 7a, which includes two parts: (a1) qnet and (a2) pnet. At time *t*, $x_{t}$ is the input network data and $x_{t}^{\prime }$ is the reconstruction of $x_{t}$. $e_{t}$ and $d_{t}$ are memory variables in GRU cells which are deterministic. $z_{t}$ is a z-space variable which is stochastic, and the edges represent the dependence between variables [93]. In Figure 7b, an example of the multivariate time series snippet with two detected malicious regions (highlighted in red) is demonstrated.
- **Convolutional neural network (CNN)** is a multi-layer perceptron ANN consisting of many hidden layers. The structure of CNN typically includes two operations: convolution and pooling. In addition, it contains fully connected layers and normalization layer. Convolution transforms input patterns via a sequence of filters to an output (usually called a feature map) that highlights the input features. Subsequently, the convolution output is processed by an activation function and then down-sampled by pooling to trim off the noisy and irrelevant data. The pooling process helps eliminate glitches in the data to improve the learning process [94,95]. The convolutional layers share multiple weights that have a few parameters which facilitates the CNN architecture’s training progress compared with other neural network models with the same number of hidden units [89].
  Wu et al. [96] proposed a CNN-based intrusion detection system. The authors used spatial and temporal network traffic features and designed a hierarchical CNN + RNN network named LuNet. The proposed system was composed of a combination of CNN and RNN models to learn from the input traffic data in synchronization with a gradually increasing granularity. Therefore, both spatial and temporal network features can be simultaneously extracted. This proposed technique does not perform well to classify attacks from Backdoors and Worms due to insufficient samples in the training dataset.
  To apply deep learning in multiple network anomaly detection datasets such as NSL-KDD, UNSW-NB15, Kyoto, WSN-DS, and CICIDS 2017, as well as provide the benchmark dataset, Vinayakumar et al. [97] proposed a framework by using a distributed deep learning model. This technique uses DNNs for handling and analyzing very large scale data in real-time network traffic. In this study, the authors provided a comparison between the deep and classical machine learning classifiers on various benchmark IDS datasets; one of the drawbacks of this research work is the time complexity of the proposed detection system which is associated with complex DNN architectures.

#### 4.3.2. Generative Deep Architectures

The generative model calculates the joint probability distributions from observed data with its classes [88], though in this section, the most popular generative techniques applied in the area of intrusion detection involve the following models.

- **Deep auto-encoder (DAE)** is basically for training efficacious coding in an unsupervised fashion and typically consists of an input layer, one (or more) hidden layer(s) and an output layer [89]. The outcome achieved in the output layer is a reconstruction of the input layer after the input nodes have been ‘squished’ via the smaller hidden layer. Therefore, DAE performs similarly to dimensionality reduction techniques such as PCA. In the case of anomaly detection, the network features which are extracted through the hidden layer could be used for training feedforward layers. The overall training of the network happens by training each autoencoder in an unsupervised manner, followed by the fine-tuning step whereby the last layer is trained by supervised network data [98].
  Muna et al. [89] proposed a detection system for network malicious activities based on auto-encoder and deep feed forward neural network. The required information and network features have been collected from TCP/IP network packets. The combination of the deep forward neural network and auto-encoder have made a solid learning algorithm to deal with both the labeled and unlabeled network features, along with both the training and testing for the evaluation process which used two popular network anomaly datasets—NSL-KDD and UNSW-NB 15. A disadvantage of this technique is the complexity of selecting appropriate parameters for the training phase while facing real-time network traffic data with a fast transmission rate. Ludwing et al. [98] proposed an intrusion detection system based on the autoencoder (AE) to classify different types of attacks. The attacks in the NSL-KDD dataset such as R2L, U2R, DoS and Probing have been classified with a total accuracy of 0.92.
- **Restricted Boltzmann machine (RBM)** is a popular deep learning technique among generative models which consist of two main architectures: deep Boltzmann machines (DBM) and the deep belief network (DBN). RBM techniques are basically applied to diminish hidden layers in the network and do not accept intra-layer connections between hidden neurons. To construct a DBN architecture, a stack of DBM should be trained by using the unlabeled data as inputs of the next layer and concatenating another layer for discrimination [89].
  Alom et al. [99] developed a deep belief network to interpret the intrusion attempts in incoming network traffic. The authors discovered the capabilities of DBN performing intrusion detection through series of experiments after training it with the NSL-KDD dataset. The trained DBN was able to detect all types of unknown attacks in the dataset and classified them into five different categories; however, in the case of unknown malicious attacks beyond those in the dataset (DoS, U2L R2L, Prode), the proposed technique would fail to identify them.
- **A deep belief network (DBN)** is a generative graphical model consisting of multiple hidden layers, with connections between the layers but not between the units within each layer. This type of deep learning architecture is a hybrid model of supervised and unsupervised learning networks. The unsupervised section was trained based on one greedy layer-by-layer connection at a time, in which the layers act as feature detectors and perform the expected classification, whereas the supervised section is one or more layers linked for classification [89].

#### 4.3.3. Challenges and Future Directions for Deep Learning Techniques

Obviously, deep learning techniques can significantly improve the NADSs’ performance effectively and efficiently, with high TPR and low FAR. Nevertheless, the network construction is often a time-consuming process to systematically specify the appropriate weights for neurons and reduce the misclassification rates. Research studies show that deep learning techniques achieve acceptable accuracy and avoid much manual work, but the structure of deep learning, particularly in the malicious feature extraction phase, requires further research to achieve a self-optimized architecture.

### 4.4. Ensemble Learning Approaches

**Figure 8:**
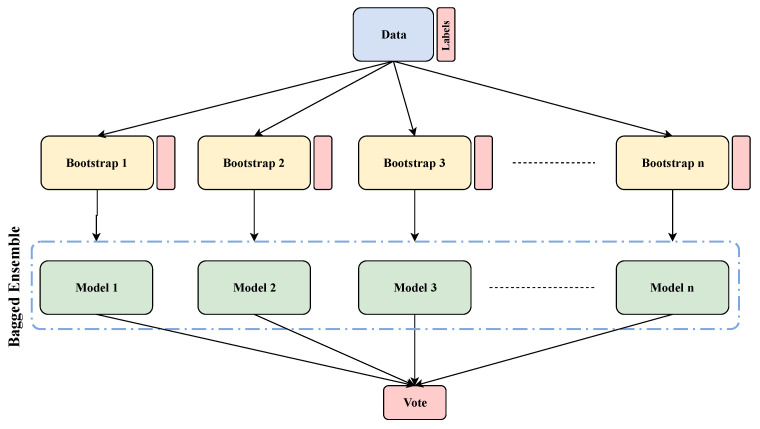
Boosting, bagging and stacking in ensemble learning approaches.

Most current NIDSs either use misuse detection or anomaly detection techniques. However, both of these techniques still face potential challenges. For instance, misuse detection techniques fail to identify anonymous intrusions, whilst on the other hand, anomaly detection approaches usually struggle with high false positive rates. In order to tackle these issues, ensemble approaches are propounded with a powerful design, exploiting features from multiple anomaly detection techniques, and the hybridization of several models enhances the performance of IDSs. Ensemble learning techniques apply multiple machine learning methods into one powerful and flexible model in order to decline variance (bagging), bias (boosting), or improve predictions (stacking) [1]. The main goal of ensemble learning is to attain an overall accuracy compared with each classifier independently. The structure of ensemble learning consists of bagging, boosting, and stack generalization/stacking as depicted in Figure 8 and explained in the following [1].

- **Bagging (bootstrap aggregation):** mostly considers homogeneous weak classifiers, trains all classifiers parallelly and independently; thereafter, it combines them based on a deterministic averaging process [1].
- **Boosting:** employs the weighted averages to construct weak classifiers into a powerful classifier. As opposed to the bagging strategy in which every classifier is run independently, boosting is all about “teamwork”. Each model that runs dictates what features the next model will focus on [1].
- **Stack generalization:** achieves the supreme overall accuracy by applying the probabilities of every model based on the specific classification method [1].

Tsai et al. [100] introduced an ensemble IDS approach based on the triangle area nearest neighbors to discover the network anomaly instances. In the first step, a K-mean clustering algorithm was applied to find the cluster centers related to the malicious classes, and in the second step, the triangle area using two cluster centers with one datum was calculated and obtained a new signature of the network datum. Finally, the KNN classifier technique was applied to categorize similar malicious activities based on the new incoming features extracted by triangle areas, though the high complexity of this proposed system in pre-processing step is a disadvantage of the detection system. Another similar work using K-means and KNN techniques is proposed in [49].

Comar et al. [101] presented a framework to detect network anomaly activities and Zero-Day malware using extracted network traffic features. The authors used supervised classification techniques (SVM and KNN) for detecting known classes with the adaptability of unsupervised learning techniques for detecting new malicious instances, though when the number of attack classes becomes very large, the detection phase becomes expensive due to the large number of hyperspheres that need to be tested. To address this issue, hierarchical multi-class learning techniques need to be incorporated with a detection system. Li et al. [46] proposed the significant permission identification (SigPID) technique that detects malicious instances based on permission usage analysis. The SigPID method utilized SVM and decision tree techniques to classify various malware and benign applications. Bamakan et al. [102] developed a multi-class technique to IDS based on a classification regression model named amp loss K-support vector. The authors utilized the K-SVCR model as a main decision engine for anomaly detection, and the SVM technique and its extensions were used to deal with the noises and outliers in the training dataset. One of the limitations of this work in its present form is facilitating the batch SVM learning over very large datasets and high network traffic. Dubey et al. [103] proposed a hybrid model for malicious detection using K-means, a back propagation neural network and naïve Bayes. Initially, the K-means clustering was applied as an unsupervised cluster analysis technique to attain the gathered data, then, the outputs were provided to the Bayesian classifier based on the probability model to obtain the most important attributes. Finally, the training and learning were accomplished by the back propagation neural network to learn the instances with minimum training cycles. The main disadvantage of this research work is the performance of attack classification via the back propagation neural network which is time consuming.

Khan et al. [104] introduced an IDS using SVM and hierarchical clustering. The authors utilized SVM to classify network-based anomalies and enhanced the training time of SVM using the hierarchical clustering technique in large scale datasets. Moreover, the authors developed a dynamically growing self-organizing tree technique to overcome the limitations of traditional hierarchical clustering algorithms such as hierarchical agglomerative clustering. Another similar work conducted in [60]. Moustafa et al. [105] proposed an ensemble IDS to detect malicious instances, specifically, Botnet attacks. The authors created statistical flow features based on significant network properties. Afterwards, developed an AdaBoost ensemble learning model based on three machine learning techniques (decision tree, naïve Bayes, ANN), to evaluate the effect of extracted features and identify network anomaly instances. Jongsuebsuk et al. [106] developed a real-time IDS using a fuzzy generic model for network attack classification. The fuzzy rule algorithm was utilized to classify the different types of attacks, while a genetic algorithm used to find an appropriate fuzzy rule and give the optimized solution. Similarly, the same author in [107] introduced another IDS based on a fuzzy genetic algorithm for various denial-of-service (DoS) attacks and Probe attacks. Damavsevivcius et al. [108] proposed an ensemble-based classification using neural networks and machine learning models for windows PE malware detection. The first phase of the classification progress was performed by a stacked ensemble of dense and convolutional neural networks (CNNs). Afterwards, the second phase was accomplished by a combined learning-based engine using 14 classifier systems called a meta-learner.

As a result, ensemble learning models are advantageous, as these techniques can deal with large scale datasets and achieve higher detection rates compared to individual techniques and perform better by combining multiple classifiers. However, it is hard to adopt a subset of stable and unbiased classification techniques to combine the models. Moreover, the greedy techniques to choose training datasets are often time-consuming for massive datasets. Among ensemble techniques, Adaboost and Stack generalization are efficacious due to the variety in predictions using multiple base level classifiers. Some other works for anomaly detection systems based on ensemble learning techniques conducted in [16,23,109,110,111,112,113]. A comparison of ensemble learning approaches on various datasets is listed in Table 3; moreover, Table 4 shows the advantages and disadvantages of machine learning approaches in the overall and the detailed comparison for all supervised learning techniques separately are listed in Table 5 and for unsupervised learning techniques in Table 6.

#### Challenges and Future Directions for Ensemble Learning Approaches

According to studies in the literature, both theoretically and empirically, it was shown that ensemble learning techniques are always preferable to single classifier techniques, with regard to classification accuracy. In terms of anomaly detection, the advantages of ensemble learning classifier techniques are particularly obvious because so many intrusions are existing in the network environments, especially intrusions in new computing technologies. Consequently, different types of detector techniques are required for identification. In addition, if one of the classifier techniques fails to identify the attack, there is still a chance to be detected in other classifier techniques. Generally, there are two types of ensemble structures with different architectures: homogeneous and heterogeneous ensembles. In homogeneous ensembles, all classifiers in the ensemble are created with a similar technique; whereas in heterogeneous ones, they are created with various classifier techniques. For example, bagging and boosting are usually utilized to create homogeneous ensembles, and stacking and voting can be applied to generate heterogeneous ensembles.

## 5. Evaluation Criteria

To accurately evaluate the performance of any IDSs, it is necessary that the system can detect the attack and normal instances (as a binary detection problem) and in case of attack recognition, the system should correctly classify the different types of attacks (as a multi-class classification problem). There are various datasets and evaluation metrics available to mathematically assess NADSs. The most popularly used datasets and evaluation metrics are discussed as follows.

### 5.1. Datasets

The dataset is an important component of any anomaly detection system to assess the efficiency and effectiveness of a detection and recognition system. The pre-processing of the captured network traffic packets in high speed traffic is a very complicated task due to the difficulty of labeling normal and malicious instances. Network traffic packages have been captured and processed in an off-line or real-time dataset by using various tools such as Gulp, Wireshark, tcptrace, etc. These raw data contain a massive variety of normal and malicious instances.

The following are recent benchmark and real-life datasets used in the domain of NADSs.

- **The UNSW-NB15 dataset** [121] was generated in 2015 at Cyber Range Lab of the Australian Center for Cyber Security (ACCS) in the University of New South Wales [121]. The UNSW-NB15 has been collected via IXIA PerfectStorm tool and consists of a hybrid of normal and synthetic contemporary attack observations, in the form of numerous patterns with normal evidences and nine groups of attacks. Backdoors, DoS, Analysis, Fuzzers, Generic, Worms, Shellcode, Reconnaissance, and Exploits are the types of attacks which are meaningfully characterized by 47 features for every attack and normal ones. A closer look at the dataset shows that 2,540,044 numbers of records (100 GB) of the raw network traffic observations were collected via different devices. This dataset consists of 700,000 samples (in total) including 677,789 normal and 22,211 malicious behaviors, respectively [122]. In the UNSW-NB15 dataset, IXIA traffic creators were connected to three different servers: servers 1 and 3 were allocated to generate normal instances and server 2 for malicious instances, while all servers are connected to two routers, and router 1 is the main router. Using router 1, all pcap files are captured to extract feature vectors. All the attack categories and the number of patterns in the training and testing subsets for each attacks are listed in Table 7b.
- **The KDD99 and NSL-KDD datasets**: the KDD99 is a benchmark dataset [123] developed in the Lincoln Laboratories of the Massachusetts Institute of Technology. The authors created a simulation involving a large variety of normal and malicious samples in the Air Force LAN environment of the US military. The analysis of the KDD99 datasets revealed some important issues that need to be addressed, so that they could negatively effect the assessment of malicious detection techniques. To settle these problems, a new benchmark dataset named NSL-KDD [124], contains the extracted records of KDD dataset was developed and has the following advantages over the first version of KDD99. The dataset is free of duplicated instances, though also the features in bot testing and training datasets are extracted from various parts of the original version of previous dataset. However, neither KDD99 nor NSL-KDD datasets are able to accurately represent network traffic data as normal and malicious instances are significantly different from contemporary network traffic [1]. All attack categories and the number of patterns in the training and testing subsets for each attack are displayed in Table 7a for the NSL-KDD dataset.
- **LITNET-2020** [125] is an annotated real-world network flow dataset for network intrusion detection and presents instances of normal and under-attack network traffic observation. The dataset consists of 85 different features utilized to classify 12 types of network attacks. The experimental analysis of this dataset conducted by two classical and four modern datasets by key features and described its advantages and limitations. The network traffic data captured over 10 months and this type of network data acquisition provides an advantage compared to the other artificial synthetically generated datasets.
- **The CAIDA dataset** [126] is a benchmark dataset which contains various types of malicious events to evaluate anomaly detection systems, particularly DoS and DDoS attacks with the footprint of the packet headers. The CAIDA DDoS 2007 dataset is more familiar in anomaly detection which contains an hour of anonymous network traffic data exclusively for DDoS attacks. However, this dataset has some disadvantages; the first one is that it does not have a ground truth for the attack observations and the second one is its pcap files which were not correctly examined to extract informative features.
- **The DEFCON dataset** [127] is another well-known dataset for the evaluation of anomaly detection systems. It consists of network traffic data generated during the capture the flag (CTF) hacking and information security competition. In CTF, students were distributed into two categories: attackers and defenders. The generated traffic during competition is very different from the actual network traffic instances, since it includes only intrusive traffic without normal traffic observations; thus, the DEFCON dataset is appropriate for assessing alert correlation techniques.
- **The UNIBS dataset,** [128] which is a real life dataset for IDSs, was obtained from the network router during 3 days at the University of Brescia, Italy. The network instances were captured from 20 different workstations using the tcpdump tool.
- **The Kyoto dataset** was conducted at Kyoto University. The dataset contains a portion of network traffic packages extracted from honeypot devices. Feature creation tools were used to extract 24 different features from the KDD99 datasets. Thereafter, the extracted features were classified into 14 conventional and 10 additional features to accurately represent network data properties. However, the main disadvantage of this dataset is the lack of labeling and describing attack behaviors.
- **The DARPA 2009 dataset and DARPA 2000 dataset** [129] are benchmark datasets which synthetically created the assessment of the network traffic data between the Internet and 16 sub-networks accumulated during ten days in November 2009. The dataset consists of SMTP, DNS, and HTTP background traffic instances and involves DoS and DDoS attack observations.
- **The CICIDS2017 dataset** [130] was created at the Canadian Institute for Cybersecurity. The CICIDS2017 consists of contemporary attack scenarios that was generated based on data profiling, which is the same as the ISCX dataset. The network traffic packages were processed using CICFlowMeter tools to extract significant features such as time stamp, protocol types, and IP addresses.
- **The ISCX-UNB dataset** [131] is a real-life dataset developed based on users profiling and descriptions of attacks. The datasets were recorded from a real-time simulation network environment during 7 days of normal and synthetic malicious data simulators. However, the authors included various multi-stage attack scenarios to enhance the number of malicious instances, but the ground truth of different types of attacks was not provided in this dataset to justify the credibility of the labeling [132].
- **The TUIDS dataset** [133] is a real-life dataset created at the University of Tezpur, India. The dataset includes different types of attack scenarios and the network traffic packets were collected using tools such as the nfdump and gulp to obtain representative features. The features are classified into basic, time, window, content and connectionless features from the preprocessed data and the corresponding labels [133].
- **The CDX dataset** [134] was created by the cyber-security team of the US military academy. The dataset was captured over a network warfare competition and contains ASNM features collected from tcpdump files of normal and malicious instances.
- **The CTU-13 dataset** [135] was captured at the CT university, including normal traffic and Botnets data instances (13 batches of various Botnet scenarios). In every scenario, a specific malicious datum was implemented using many protocols and executed actions.
- **NGIDS-DS dataset** [59] was created at the New South Wales university in Australia to investigate Linux Host-based IDSs. The dataset consists of a variety of kinds of malicious and normal instances generated using feature creation tools such as the IXIA perfect-storm and stored in different CSV files.
- **The LBNL dataset** [136] is a benchmark dataset developed at the Lawrence Berkeley National Laboratory (LBNL). Two different routers have been used at the LBNL to collect network traffic packets and include about 1000 host systems for approximately 100 h.
- **The ADFA dataset** [137] was created at the New South Wales university in Australia to investigate Linux and Windows host-based IDSs. In the training phase, the system call traces greater than 300 bytes to 6 kB were neglected and also, in the evaluation phase, the traces outside of the bound were omitted.

To recommend a suitable dataset for a researcher in the area of attack classification and malicious behavior detection, two popular datasets (the NSL-KDD dataset in Table 7a and UNSW-NB15 dataset in Table 7b) and the corresponding attack categories are listed in Table 7. Overall, Table 8 represents a comparison of 10 well-known datasets from the literature, and the comparison results show that the UNSW-NB15 dataset meets the important requirements of a reliable dataset to evaluate NADSs.

**Table 7:** Dataset distribution for two popular datasets.

	(a) The NSL-KDD Dataset	
**Category**	**Training Data**	**Testing Data**
DoS	45,927	7458
R2L	995	2887
U2R	52	67
Probe	11,656	2422
Normal	67,343	9710
Total Records	125,973	22,544
	**(b) The UNSW-NB15 Dataset**	
**Category**	**Training Data**	**Testing Data**
Normal	56,000	37,000
Backdoor	1746	583
Analysis	2000	677
DoS	12,264	4089
Generic	40,000	18,871
Shellcode	1133	378
Reconnaissance	10,491	3496
Fuzzers	18,184	6062
Exploits	33,393	11,132
Worms	130	44
Total Records	175,341	82,332

**Table 8:** Comparison of 10 popular datasets for NADSs.

Datasets	Year	U	V	W	X	Y	Z
UNSW-NB15 [121]	2015	Yes	Yes	Yes	Yes	Yes	Yes
KDD99 and NSL-KDD [123]	1999	Yes	No	Yes	Yes	Yes	Yes
NGIDS-DS [59]	2017	Yes	Yes	Yes	Yes	Yes	No
CAIDA [126]	2007	Yes	Yes	No	No	No	No
DEFCON [127]	2002	No	No	Yes	No	Yes	Yes
UNIBS [128]	2009	Yes	Yes	Yes	Yes	No	Yes
DARPA [129]	2009	Yes	Yes	No	No	Yes	Yes
ISCX and CICDS [131]	2017	Yes	Yes	Yes	Yes	Yes	Yes
TUIDS [133]	2012	Yes	Yes	Yes	Yes	Yes	Yes
LBNL [136]	2016	No	Yes	No	No	Yes	No

**U**: (whether the dataset provided realistic network configuration information); **V**: (whether the dataset provided realistic network traffic); **W**: (whether the dataset captured total interaction); **X**: (whether the observations labeled accurately); **Y**: (whether the dataset provided many malicious scenarios); **Z**: (whether full packets were captured).

### 5.2. Evaluation Metrics

The performance of methods based on machine learning techniques and distance-based measurement methods depend on calculating a confusion matrix to assess the performance and effectiveness of the decision engine; in a binary classification normal observation (class 1) discriminated against malicious ones (class 2) and the corresponding confusion matrix depicted in Table 9.

The terms true positive (TP), true negative (TN), false negative (FN) and false positive (FP) are popular evaluation elements to produce evaluation measurements such as false positive rate (FPR), true positive rate (TPR), precision, recall and F-measure. In anomaly detection scenarios, these terms are utilized to produce the following malicious behavior evaluation metrics described as follows:

- **Confusion matrix:** compares the predicted class labels against the actual ones. The diagonal cells (TNs and TPs) represent the correct predicted classes and the other sides represent FNs and FPs. The size of the confusion matrix depends on the number of predefined classes. The confusion matrix for IDS can be binary detection, as shown in Table 9, which is a 2-by-2 matrix to classify malicious instances against normal ones or a multi-class classification anomaly recognition to recognize the type of network anomalies (attacks).
- **True positive rate (TPR)** is the proportion of malicious observations correctly detected over the total number of malicious observations in the testing dataset.
- **True negative rate (TNR)** is the proportion of malicious observations wrongly detected as normal over the total number of normal observations in the testing dataset.
- **False positive rate (FPR)** is the proportion of normal observations wrongly detected as malicious over the total number of normal observations in the testing dataset. The equation below describes how *FPR* is computed:
  (3) $$\begin{array}{cc} FPR & =\frac{\left(FP\right)}{\left(TN)+(FP\right)} \end{array}$$
- **False negative rate (FNR)** is the proportion of normal observations classified as normal over the total number of malicious observations in the testing dataset.
  (4) $$\begin{array}{cc} FNR & =\frac{\left(FN\right)}{\left(FN)+(TP\right)} \end{array}$$
- **Precision** is the proportion of malicious observations correctly detected over the total number of detected observations in the testing dataset:
  (5) $$\begin{array}{cc} Precision & =\frac{\left(TP\right)}{\left(TP)+(FP\right)} \end{array}$$
- **Recall** is the proportion of malicious observations correctly detected over the total number of malicious observations in the testing dataset:
  (6) $$\begin{array}{cc} Recall & =\frac{\left(TP\right)}{\left(TP)+(FN\right)} \end{array}$$
- **F-measure** mixes the properties of both precision and recall measures and is a harmonious mean of these two metrics:
  (7) $$\begin{array}{cc} F-measure & =2*\frac{\left(Precision)*(Recall\right)}{\left(Precision)+(Recall\right)} \end{array}$$
  *F-measure* is a powerful measure for anomaly detection when the problem contains unbalanced classes or target values.
- **Overall accuracy** basically assesses how accurately an anomaly detection system works by measuring the percentage of correctly classified and miss-classified patterns. If the accuracy of a system is 80%, this means the system correctly detected 80 patterns out of 100 to their actual classes. The below equation describes how the overall accuracy is computed:
  (8) $$\begin{array}{cc} Overall\;Accuracy & =\frac{\left(TP)+(TN\right)}{\left(TP)+(TN)+(FP)+(FN\right)} \end{array}$$
- **Receiver operating characteristics (ROC) curve** is originally derived from signal processing theory. In network anomaly detection, ROC curves visualize the relation between significant rates such as *FP* and *TP* rates of a classifier system and also to figure out the accuracy performance between two or more classifier systems.
- **Matthews correlation coefficient (Mcc)** can only be employed in binary malicious behavior detection systems in which the users’ behaviors are classified as either normal or attack [4]:
  (9) $$\begin{array}{cc} Mcc & =\frac{\left(TP*TN)-(FP*FN\right)}{\sqrt{\left(TP+FP)(TP+FN)(TN+FP)(TN+FN\right)}} \end{array}$$

Anomaly detection systems and broadly IDS techniques are evaluated to assess to what extent these techniques are precise in detecting malicious observations. An ideal detection system that achieves a 100% DR with 0% FPR indicates that all malicious activities are correctly identified without any miss-detection. However, such ideal detection systems are experimentally not achievable or very hard to achieve in a real-time network traffic environment due to the complexity of traffic packages, and the large size and speed of contemporary network systems. Sensitivity (also called TPR or recall) is more advantageous when the designed system is preserved at the cost of a high FPR and FNR. On the other hand, specificity (also called TNR) is more suitable when the accuracy of a system is very low. The accuracy measure is a useful metric whenever intrusion detection data are balanced, however, in real network traffic data, the normal instances are significantly more than the malicious ones.

## 6. Challenges and Future Directions

Contemporary malware detection systems are often insufficient for the new generation of malware, because such malicious software are created based on novel attacking technologies. In this context, users’ observations including both normal and malicious instances, and need to be carefully characterized and represented using the extracted packets throughout a network. The responsibility of a malware analysis system is not only to detect observations as malicious or normal, though the system might be improved to recognize the type of abnormality that malware may perform (attack types). The authors believe that an integration of traditional IDSs with recognition-based techniques can expand the system into a new intrusion detection and recognition system. This supports that the system can detect all malicious observations, and then classify the type of abnormality to accomplish the appropriate response according to the attack nature [14].

As a result, existing studies in the area of NIDSs, show that it is still very hard to develop a new NIDS to ensure three important properties such as robustness, scalability and high performance in emerging technologies, to effectively and efficiently prevent any types of malicious activities. In addition, experimental works show that locating the place of NIDS and the best configuration for deployment within shared environments in new computing technologies such as Cloud, edge, Fog computing and IoT, with various stakeholders is also a very challenging task [132,138]. Some of the important issues with respect to different phases of NADSs are sorted out as main challenges in the following.

### 6.1. Network Feature Selection and Extraction

In feature extraction phase, the dynamic processing and static processing methods have some superiority and drawbacks. In real-time detection systems, it is preferable to use static processing at the beginning until approximately 80% of network data instances are well represented by using static features. In case of any probable problem in static feature representation, the features can be represented using dynamic processing. In addition, recent features, e.g., file-to-file relation graphs, contain informative data about the network properties.

### 6.2. Detection and Recognition Machine

- Based on various IDS datasets with multiple feature extraction tools, there is no single machine learning technique which always performs well. Moreover, the performance of anomaly detection techniques significantly depends on how to extract informative features that can discriminate malicious instances against normal ones via detection engines.
- Generally, ensemble learning classifiers can always perform better compared with individual learning classifiers and achieve higher accuracy. In real-time network anomaly detection systems, a successful malicious detection scheme needs to apply various diverse classifiers over different kinds of feature representations.
- Another challenging task in ensemble learning architectures is how to select a suitable number of unbiased and non-correlated classifiers among the various supervised and/or unsupervised techniques to create an appropriate ensemble system for NADS.
- Since the properties of malware keep changing gradually, accordingly, the malicious detection and recognition techniques should be improved and updated with the contemporary anomalies encountered in the local network or the Internet.
- Cyber espionage attacks detection using traditional data extraction tools, which has become one of the main challenges in IoT networks [25]. It is very hard to identify these kinds of malicious activities through traditional NADSs, because the profiles of normal instances for telemetry data of IoT sensors and network traffics still need to be created. A combination of deep learning and mixture algorithms can improve the NADSs performance.

### 6.3. Datasets

- The dynamic updating of NADS datasets is a very important issue. The existing instances in the database should be updated as soon as a new malicious observation is detected by NADS.
- The availability of an unbiased NADS dataset is another concern for training and validating NADS models. Briefly, normal instances are significantly larger then malicious ones in existing publicly available NADS datasets. For example, the KDD99 dataset contains only four types of attacks and a huge amount of normal instances; similarly, the UNSW-NB15 dataset contains nine groups of attacks (total of 22,211 malicious instances) against 677,789 normal instances. Therefore, a benchmark and unbiased network anomaly dataset for evaluating anomaly detection techniques is required. Other datasets suffer from wrong labeling, less attack variety, and the incompleteness of network data—which is not containing both headers and payloads. To create a new IDS dataset for future works realistic environments is required that contains mixture of various normal and contemporary attack scenarios in new computing technologies (e.g., zero-day attacks). In addition, the ground truth that contains attack specifications needs to be created to trust the datasets credibility while assessing new NADSs.
- It is usually better to provide sufficient features in the training dataset with balanced distributions for normal and malicious instances to achieve the best reliable performance.

### 6.4. Real-Time Response

- Real-time malicious detection is an extremely challenging part of a NADSs. The first one is related to a pre-processing step in which the network traffic packages always contain a set of irrelevant and duplicated instances that need to be carefully and accurately eliminated. The second one is the structure of detection and recognition techniques which need to be dynamically adopted for existing and zero-day attacks. The above reasons increase the pre-processing and detection time if not carefully addressed.
- Runtime limitation is a significant issue for anomaly detection systems. Without escaping any network packets, a real-time anomaly detection should be ideally suited and be capable to capture and extract every packet.

### 6.5. Managing False Alarm (False Positive/Negative Errors)

FPR and FNR errors happen when there is miss-classification between normal and malicious behaviors; in this case, normal instances may fall in an anomaly region and conversely, a malicious one in the normal region. This is a very challenging and complex task almost in all detection techniques. Creating a comprehensive profile that comprises all normal behaviors is extremely difficult because the discrimination boundary between legitimate and malicious instances is not always accurate; some recent attacks intelligently mimic normal behaviors to deceive detection systems.

### 6.6. Adaptively and Scalability

New computing technologies such as IoT, Cloud/Fog computing paradigms and deployed devices are expected to handle a big amount of data in high-speed networks that exchange high data rates in real time; consequently, the architecture of new NADSs should be self adaptive and scalable to monitor the large size and high speeds of current network traffics. To this end, a collaborative NADS is required to process multiple network nodes and concatenate its data to detect and recognize any malicious observation [22,70].

## 7. Conclusions

This paper discussed the state-of-art in the recent IDSs and applications, particularly anomaly detection systems. Although IDSs play a significant role in cyber-security systems and applications, this technology still faces challenges for designing a real-time, reliable and compatible system for online applications. Generally, NADS is assessed based on network anomaly datasets, including a mixture of recent normal and malicious instances, which reflects the efficiency of the detection system. In order to extract and prepare the informative features from these datasets, data pre-processing methods including feature creation, reduction, transformation, and normalization have been discussed to provide machine learning techniques with appropriate features.

The main part of a NADS is the detection and recognition of malicious observations; therefore, in this paper, the authors comprehensively discussed different types of machine learning systems and applications to detect malicious instances against normal ones; various machine learning methods based on supervised learning, unsupervised learning and new ensemble and deep learning techniques have been discussed to comparatively show their superiorities and limitations in terms of designing an effective network anomaly detection system. In addition, several evaluation metrics for measuring the performance of anomaly detection techniques have been discussed. A brief description of multiple network anomaly datasets is also provided. For the future research directions, it is possible to investigate the detection and recognition of zero-day attacks and as well as other deep learning models without pattern labeling and feature extraction formalities in high-speed network traffic data.

Given a comprehensive learning-based anomaly detection architecture, followed by various properties for each phase discussed in this survey article, the authors believe it is appropriate for researchers working in the domain to meet all of the key criteria for designing and developing new network anomaly detection systems.

## Figures and Tables

**Table 1:** A comparison of the proposed survey against existing survey articles based on the main phases proposed in Figure 1.

NADS Aspects	[4]	[5]	[6]	[12]	[7]	[8]	[13]	[9]	[10]	[11]	Proposed Survey
Network Data Pre-Processing	×	✓	✓	×	✓	✓	✓	✓	✓	×	✓
Supervised Learning Approaches	✓	✓	✓	✓	✓	×	✓	×	✓	✓	✓
Unsupervised Learning Approaches	✓	✓	×	✓	✓	✓	✓	✓	✓	✓	✓
Deep Learning Approaches	×	✓	×	×	✓	×	✓	×	×	×	✓
Ensemble Learning Approaches	×	×	✓	×	×	✓	×	×	✓	×	✓
Datasets Discussion and Comparison	×	×	×	×	×	×	×	×	×	✓	✓
Evaluation Criteria	✓	×	✓	×	✓	×	×	×	×	✓	✓

**Table 2:** Features and categories in the UNSW-NB15 datasets [14].

Feature Type	Feature Number	Total
Integer	2,4,8,9,10,11,12,13,17,18,19,20,21,22,23,24,25,26,37,38,40,41,42,43,44,45,46,47	28
Nominal	1,3,5,6,14	5
Timestamp	29,30	2
Float	7,15,16,27,28,31,32,33,34,35	10
Binary	36,39	2

**Table 3:** Comparison of ensemble learning approaches on various datasets.

Used Algorithms	Authors	Objective(s)	FS	Accuracy (%)	FAR (%)	Datasets	Detected Attack(s)	Ref
K-means + KNN	Tsai et al.	IDS	Yes	93.55	4.79	KDD-Cup’99	DoS, U2L R2L, Prode	[100]
	Bar et al.	Encrypted traffic classification	Yes	95.4	-	Generated records	Malicious instances	[49]
	Lin et al.	IDS	Yes	99.46	2.95	KDD-Cup 99	DoS, U2L R2L, Prode	[50]
SVM + KNN	Comar et al.	Zero-day malware detection	Yes	90	10	Commercial IDS/IPS	High-risk malwares	[101]
SVM + kNN + PSO	Aburomman et al.	IDS	Yes	88.44	-	KDD-Cup 99	DoS, U2L R2L, Prode	[113]
SVM + DT	Li et al.	Android malware detection	Yes	93.62	2.36	Generated dataset	-	[46]
K-Support Vector Classification-Regression	Bamakan et al.	IDS	Yes	98.68	0.86	NSL-KDD + UNSW-NB15	All attacks in Table 7b	[102]
PCA Filtering + Probabilistic SOM	Hoz et al.	IDS	Yes	88	-	KDD99	Anomalous connections	[111]
K-Means + NB + BNN	Dubey et al.	IDS	No	99.9	0.1	KDD cup99	DoS, U2R, R2L, probe	[103]
Density Based Clustering + GMM	Gruhl et al.	IDS	No	98.4	0.9	-	-	[114]
HC + SVM	Horng et al.	IDS	Yes	95.72	-	KDD Cup 1999	DoS, U2R, R2L, probe	[60]
	Khan et al.	IDS	No	69.8	37.8	1998 DARPA	DoS, U2R, R2L, probe	[104]
RF + AODE	Jabbar et al.	IDS	Yes	90.51	14	Kyoto	-	[42]
DT + NB + ANN	Moustafa et al.	IDS	Yes	95.25	0.01	UNSW-NB15 and NIMS Botnet	Botnet, all attacks in Table 7b	[105]
NB + KNN	Pajouh et al.	NADS	Yes	84.86	4.86	NSL-KDD	DoS, U2R, R2L, probe	[23]
Multivariate Correlations + Triangle Area	Tan et al.	DoS attack detection	Yes	99.93	2.64	KDD Cup 99	DoS	[109]
SVM + DT + KNN	Mohaisen et al.	Malware classification	Yes	98	-	AutoMal	ZAccess, Ramnit, FakeAV, Autorun, TDSS, Bredolab, Virut	[110]
	Santos et al.	Unknown malware detection	Yes	94.5	5.5	VxHeavens	Malware families	[115]
SVM + RF + DT	Islam et al.	Malware classification	Yes	97.055	0.055	Generated datasets	-	[116]
SVM + KNN + NB + RF	Wang et al.	Malicious apps detection	No	99.39	-	-	Android malicious instances	[117]
FL + ES	Liao et al.	Network forensics	Yes	91.5	-	DARPA 2000	DDoS, DARPA attacks	[16]
GMMs + PSO + SVM	Hu et al.	IDS	Yes	99.99	1.35	KDD CUP 1999	DoS, U2R, R2L, probe	[112]
FL + GA	Jongsuebsuk et al.	IDS	Yes	97.5	13	KDD99	Various DoS and Probe attacks	[106]
	Jongsuebsuk et al.	IDS	Yes	97	-	KDD99	DoS, Probe	[107]
	Chadha et al.	IDS	Yes	94.6	-	DARPA-KDD99	DoS, U2R, R2L, probe	[118]

**FS**: feature selection; **FAR**: false alarm rate; **SVM**: support vector machine; **KNN**: K-nearest neighbors; **PSO**: particle swarm optimization; **DT**: decision tree; **PCA**: principal component analysis; **SOM**: self-organizing map; **GMM**: Gaussian mixture model; **AODE**: average one-dependence estimator; **BNN**: back-propagation neural network; **HC**: hierarchical clustering; **NB**: naïve Bayes; **AODE**: average one-dependence estimator; **RF**: random forest; **FL**: fuzzy logic; **ES**: expert system; **GA**: genetic algorithm; **IDS**: intrusion detection system; **NADS**: network anomaly detection system.

**Table 4:** Comparison of machine learning mechanisms.

Technique	Advantages	Disadvantages
Supervised Learning Approaches	Techniques are flexible for training and testing.	The techniques are very much relying on the presumptions.
	Achieve high detection accuracy for known attacks based on appropriated threshold.	Need more patterns compare with other methods.
	The techniques are able to update implementation strategies with the concatenation of new data.	The techniques fail to identify unknown attacks until similar training data are fed.
Unsupervised Learning Approaches	In case of clustering approach, such as K-mean, if k value is determined, then the remaining process is easy.	Most approaches have been developed for the clustering of continuous features only.
	Clustering techniques are advantageous for quick response generation.	In anomaly detection systems based on clustering, an initial assumption is assigning a big cluster to the normal instances and smaller clusters to malicious instances. In the absence of this assumption, it is hard to assess the technique.
	In the case of a large training dataset, it is better to split it into similar classes to efficiently detect malicious instances, because it decreases the computational complexity.	Using unsuitable proximity measures often reduces the detection rate.
	The techniques provide a trustworthy performance in comparison to supervised or statistical approaches.	It is usually time-consuming to dynamically update the profiles.
	The techniques can identify outliers easily in small datasets.	Unsupervised techniques often utilize both clustering and outlier detection and it produces higher complexity compared with other methods.
	The techniques can detect bursty and isolated attacks.	The detection parameters are highly dependent on these techniques.
Ensemble Learning Approaches	The ensemble classifier techniques perform better by combining multiple classifiers whenever the individual classifiers are weak.	Subset selection among unbiased classifiers is a difficult task.
	Appropriated for large scale datasets.	The greedy approach for choosing subsets is often time-consuming for large scale datasets.
	Ensemble techniques use a set controlling parameters that are comprehensive and can be easily adjusted.	Real-time performance is hard to achieve.
	Adaboost and Stack generalization are efficacious due to the variety in predictions using multiple base level classifiers.	Lack of suitable hybridization often faces high computational costs.

**Table 5:** Comparison of supervised learning approaches on various datasets.

Used Algorithms	Authors	Objective(s)	FS	Accuracy (%)	FAR (%)	Datasets	Detected Attacks	Ref
Support Vector Machine (SVM)	Prabaharan et al.	MDS	Yes	72.75	-	-	Drive-by-download	[45]
	Ambusaidi et al.	IDS	Yes	99.79 (KDD Cup 99 dataset)	13 (KDD Cup 99 dataset)	KDD Cup 99, NSL-KDD, Kyoto	DoS, Probe, R2L, U2L	[43]
	Wagner et al.	NADS	No	93.4	1	Lincoln	Zero-day attacks	[44]
	Kang et al.	IDS	Yes	96.9	7.7	DARPA	DOS, R2L, U2R, PROBE	[34]
	Perdisci p et al.	NADS	Yes	97.6	2.4	Simulated dataset	Payload-based anomalies	[35]
K-Nearest Neighbors (KNN)	Alazab	Malicious codes detection	Yes	94	-	Honeynet project Datasets	Malware and benign	[48]
	Bar et al.	Anomaly traffic detection	No	99.1	-	-	Payload-based anomalies	[49]
	Zargar et al.	IDS	Yes	99.01	17	DARPA 1998	DOS, R2L, U2R, Probe	[33]
	Lin et al.	IDS	Yes	80.6	11.4	KDD-Cup 99	DOS, R2L, U2R, Probe	[50]
Bayesian Networks	Jing et al.	IDS	Yes	-	-	DARPA 1998	Intrusions using system call logs	[52]
	Hesham Altwaijry	IDS	Yes	96	3.15	KDD-99 Dataset	DOS, Probe, U2R and R2L	[53]
	Moustafa et al.	Attack detection	Yes	95.98	4.02	UNSW-NB15	All attacks in Table 7	[54]
Decision Tree	Singh et al.	Botnet detection	Yes	99.8	3	CAIDA	Botnets	[41]
	Narouei et al.	Malware detection	Yes	97.8 (Depth 6)	8.3	Nappa	Malware and benign programs	[40]
	Jabbar et al.	IDS	Yes	82.5	17.3	Kyoto	Attacks in Kyoto dataset	[42]
Fuzzy Technique	Dickerson et al.	IDS	Yes	-	-	-	Malicious instances	[57]
	Haider et al.	IDS	Yes	92.8	8.1	NGIDS-DS	DoS and DDoS	[59]

**FS**: feature selection; **FAR**: false alarm rate; **MDS**: malicious detection system; **IDS**: intrusion detection system; **NADS**: network anomaly detection system.

**Table 6:** Comparison of unsupervised learning approaches on various datasets.

Used Algorithms	Authors	Objective(s)	FS	Accuracy (%)	FAR (%)	Datasets	Detected Attacks	Ref
K-means Clustering	Nguyen et al.	IDS	No	90.22	2.75	KDD99	DOS, R2L, U2R, Probe	[66]
	Lee et al.	Attack detection	Yes	-	-	DARPA 2000	DDoS	[62]
	Li	NADS	Yes	92.3	5.81	KDD CUP 1999	DoS, U2R, R2L, Probe	[61]
	Costa et al.	IDS	No	-	-	KddCup, NSL-Kdd and Netflow	Different attacks	[63]
	Jadhav et al.	IDS	No	90	-	-	Malicious instances	[64]
HC	Horng et al.	IDS	Yes	95.72	7	KDD Cup 99	DoS, U2R, R2L, Probe	[60]
GMM	Moustafa et al.	Web application attacks	Yes	95.68	4.32	UNSW-NB15	All attacks in Table 7	[67]
	Fan et al.	NADS	Yes	79.45	13.91	KDD Cup 1999 and Kyoto	DoS, U2R, R2L, Probe	[68]
	Moustafa et al.	NADS	Yes	96.7	3.5	UNSW-NB15	All attacks in Table 7	[20]
PCA	Han et al.	IDS	Yes	80.31	-	KDD CUP 99	DoS, U2L R2L, Prode	[73]
	Bhagoji et al.	Malicious detection	No	97.52	-	MNIST	Vanilla, Strategic attacks	[74]
	Jamdagni et al.	IDS	Yes	99	-	DARPA 99 and GATECH	Payload-based attacks	[37]
	Ding et al.	NADS	Yes	-	-	-	Anomalies network traffic	[75]
HMMs	Moustafa et al.	Malicious detection	Yes	98.45	2.21	UNSW-NB15	All attacks in Table 7	[21]
	Lin et al.	Virus detection	Yes	-	-	-	-	[77]
GA	Folino et al.	IDS	No	95	-	KDD Cup 1999	DoS, U2L R2L, Prode	[86]
	Hasan et al.	IDS	Yes	-	-	-	Malicious instances	[119]
	Pillai et al.	IDS	Yes	-	-	Created dataset	Port scanning attacks	[87]
ANN	Hawkins et al.	Outlier detection	Yes	-	-	KDD Cup 1999	DoS, U2L R2L, Prode	[80]
	Saber et al.	IDS	No	80.05	-	KDD99	DoS, U2L R2L, Prode	[78]
	Jirapummin et al.	IDS	No	90	5	KDD cup 1999	Neptune, Port Sweep, Satan	[81]
	Ghosh et al.	IDS	Yes	84	7	DARPA	U2R, R2L	[82]
	Saurabh et al.	NADS	No	82.72	7	KDD Cup 99	DoS, U2L R2L, Prode	[84]
	Rabbani et al.	NADS	Yes	97.5	3.6	UNSW-NB15	All attacks in Table 7	[14]
SOM	Ramadas et al.	NADS	Yes	76.06	-	-	-	[79]
Deep Learning	Ludwing et al.	IDS	Yes	92.5	5.7	NSL-KDD	DoS, U2L R2L, Prode	[98]
	Bontemps et al.	NADS	No	86	0	KDD99	DoS	[90]
	Shone et al.	IDS	No	97.85	2.15	KDDCup99 and NSL-KDD	DoS, U2L R2L, Prode	[91]
	Maya et al.	NADS	No	-	-	Artificial datasets	Anomalies	[92]
	Wu et al.	IDS	No	82.78	4.72	NSLKDD and UNSW-NB15	All attacks in Table 7	[96]
	Yin et al.	NIDS	Yes	81.29	1.27	KDDTest	DoS, U2L R2L, Prode	[120]
	Muna et al.	Malicious detection	No	98.4, 92.5	1.8, 8.2	NSL-KDD and UNSW-NB 15	All attacks in Table 7a,b	[89]
	Alom et al.	IDS	Yes	97.5	-	NSL-KDD	DoS, U2L R2L, Prode	[99]

**FS**: feature selection; **FAR**: false alarm rate; **HC**: hierarchical clustering; **GMM**: Gaussian mixture models; **PCA**: principal component analysis; **HMMs**: hidden Markov models; **ANN**: artificial neural network; **GA**: genetic algorithm; **SOM**: self-organizing maps; **IDS**: intrusion detection system; **NADS**: network anomaly detection system.

**Table 9:** Confusion matrix for binary anomaly (attack and normal) detection.

		Actual	
		Positive (Attacks Classes)	Negative (Normal Class)
Predicted	Positive (Attacks classes)	TP	FP
	Negative (Normal class)	FN	TN

## Data Availability

Data sharing not applicable.
